# Chikungunya virus infection disrupts lymph node lymphatic endothelial cell composition and function via MARCO

**DOI:** 10.1172/jci.insight.176537

**Published:** 2024-01-09

**Authors:** Cormac J. Lucas, Ryan M. Sheridan, Glennys V. Reynoso, Bennett J. Davenport, Mary K. McCarthy, Aspen Martin, Jay R. Hesselberth, Heather D. Hickman, Beth A.J. Tamburini, Thomas E. Morrison

**Affiliations:** 1 Department of Immunology & Microbiology and; 2 RNA Bioscience Initiative, University of Colorado School of Medicine, Aurora, Colorado, USA.; 3 Viral Immunity & Pathogenesis Unit, Laboratory of Clinical Immunology & Microbiology, National Institutes of Allergy & Infectious Disease, NIH, Bethesda, Maryland, USA.; 4 Department of Biochemistry & Molecular Genetics and; 5 Division of Gastroenterology and Hepatology, Department of Medicine, University of Colorado School of Medicine, Aurora, Colorado, USA.

**Keywords:** Immunology, Virology, Endothelial cells

## Abstract

Infection with chikungunya virus (CHIKV) causes disruption of draining lymph node (dLN) organization, including paracortical relocalization of B cells, loss of the B cell–T cell border, and lymphocyte depletion that is associated with infiltration of the LN with inflammatory myeloid cells. Here, we found that, during the first 24 hours of infection, CHIKV RNA accumulated in MARCO-expressing lymphatic endothelial cells (LECs) in both the floor and medullary LN sinuses. The accumulation of viral RNA in the LN was associated with a switch to an antiviral and inflammatory gene expression program across LN stromal cells, and this inflammatory response — including recruitment of myeloid cells to the LN — was accelerated by CHIKV-MARCO interactions. As CHIKV infection progressed, both floor and medullary LECs diminished in number, suggesting further functional impairment of the LN by infection. Consistent with this idea, antigen acquisition by LECs, a key function of LN LECs during infection and immunization, was reduced during pathogenic CHIKV infection.

## Abstract 2

<p>Chikungunya virus disrupts the organization and function of lymph nodes by targeting distinct populations of lymphatic endothelial cells via the scavenger receptor MARCO.</p>

## Introduction

Chikungunya virus (CHIKV), a mosquito-transmitted arthritogenic alphavirus, remains a persistent threat to global health 10 years after spreading to the Americas and 20 years since epidemic-level outbreaks occurred in the Indian Ocean region (1, 2). CHIKV disease typically presents with acute fever, rash, and severe arthralgia, and up to 60% of patients remain chronically afflicted with arthritis and arthralgia for months to years after infection (2, 3), constituting a high socioeconomic burden (3). Studies in both animal models (4–8) and patients (9, 10) suggest that chronic CHIKV disease is associated with persistence of viral RNA and antigen in cells within joint-associated tissue.

In prior studies using an immunocompetent mouse model of CHIKV infection, we found that CHIKV evades the B cell response to establish viral persistence in joint-associated tissues. These studies reveal that peripheral lymph nodes are important for the generation of the CHIKV-specific B cell response and control of CHIKV infection (6, 7, 11, 12). Closer examination of the lymph node response during CHIKV infection revealed that WT CHIKV infection disrupts the structure and function of the draining lymph node (dLN), the first secondary lymphoid organ to encounter virus following infection (13), in contrast to infection with the attenuated CHIKV 181/25 strain, which does not establish persistent infection in mice (11). This disruption of dLN organization is mediated by an early influx of inflammatory myeloid cells that contribute to diminished lymphocyte recruitment and retention, disruption of the B cell–T cell border, relocalization of B cells, and poor germinal center formation (12, 13). However, the specific cell types that interact with CHIKV and promote inflammation in the dLN remain to be elucidated.

The architecture and cellular organization of LNs are essential for the development of effective immune responses against viral antigens (14). The organization of LNs is coordinated by lymph node stromal cells (LNSCs), including fibroblastic reticular cells (FRCs), lymphatic endothelial cells (LECs), and blood endothelial cells (BECs). These cell populations provide a physical scaffold for immune cell migration and produce signals to regulate migration, adhesion, localization, function, and survival of hematopoietic cells. FRCs and LECs can be grouped into distinct subsets based on transcriptional signatures and regional localization within the LN (15–20). LECs are among the first cells in the LN to encounter viruses, cells, and antigens draining into the LN via the afferent lymphatics (21–23). In addition, LN LECs play an active role in enhancing immune responses through internalization and retention of antigen during vaccination and infection (24–26).

Recently, we found that CHIKV dissemination within an infected host is restricted by LNs and the scavenger receptor macrophage receptor with collagenous structure (MARCO), and that viral particles colocalize with MARCO^+^ LECs in the dLN (27). Single-cell RNA-Seq (scRNA-Seq) of LNSCs confirmed that viral RNA in the dLN accumulated largely in a subset of LECs that express MARCO, termed MARCO^+^ LECs (27). Since LECs are important regulators of LN tissue organization and function (28–31), we hypothesized that the interaction between CHIKV and MARCO^+^ LECs promotes LN inflammation previously shown to impair B cell responses during CHIKV infection.

In this study, using scRNA-Seq, we identified MARCO-expressing floor LECs that line the subcapsular sinus (SCS) as a site of early viral RNA accumulation. These findings, together with our prior observations that CHIKV RNA subsequently accumulates in MARCO^+^ LECs that line the medullary sinuses, suggest that CHIKV targets multiple subsets of MARCO-expressing LECs in the LN. CHIKV infection caused dramatic alterations to the gene expression program of LNSCs, including LECs and other LNSC subtypes, that were characterized by an inflammatory gene transcriptional response, and this early inflammatory response was accelerated by CHIKV-MARCO interactions. Quantification of LN LEC subsets throughout the acute phase of CHIKV infection revealed reduced numbers of both floor and medullary LECs at later times after infection, and these reductions were MARCO dependent. Evaluation of LN LEC function during CHIKV infection revealed that WT, but not attenuated, CHIKV infection impairs antigen acquisition by LN LECs in a MARCO-dependent manner. Collectively, these findings identify a role for the scavenger receptor MARCO in regulation of LN inflammation and support a model by which CHIKV targeting of MARCO-expressing LECs initiates an inflammatory response that rewires the transcriptional program of LNSCs, alters the composition of specialized LEC subtypes, and impairs known LEC functions.

## Results

### CHIKV RNA accumulates in MARCO-expressing LN LECs.

**Figure 1:**
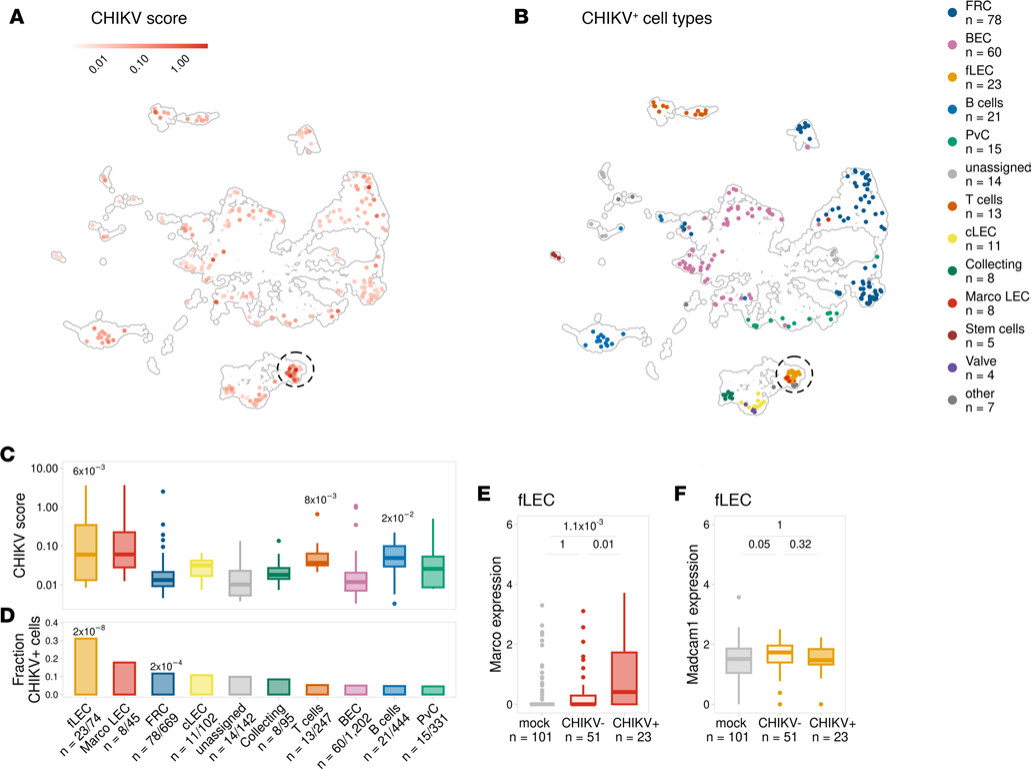
CHIKV RNA accumulates in MARCO-expressing floor LECs in the dLN. (**A**–**F**) WT mice were inoculated with PBS (mock, *n* = 2) or 1 × 10^3^ PFU of CHIKV (*n* = 2) in the footpad. At 8 hours after infection, the dLN was collected and enzymatically digested into a single-cell suspension. Cells were enriched for CD45^–^ cells and analyzed by scRNA-Seq. (**A**) UMAP shows CHIKV score, calculated as the fraction of total reads that align to the CHIKV genome for each cell from the CHIKV-enriched libraries. (**B**) UMAP shows CHIKV^+^ cells. (**C**) CHIKV score is shown for CHIKV^+^ cells for cell types with > 40 total cells and > 3 CHIKV^+^ cells. *P* values were calculated using a 1-sided Wilcoxon rank sum test with Bonferroni correction comparing each cell type with all other CHIKV^+^ cells. Only adjusted *P* < 0.05 are shown. (**D**) The fraction of cells identified as CHIKV^+^ is shown for each cell type in **C**. *P* values were calculated using a 1-sided hypergeometric test with Bonferroni correction. Labels show the number of CHIKV^+^ cells/total cells. Only adjusted *P* < 0.05 are shown. (**E** and **F**) *Marco* and *Madcam1* expression for floor LECs (fLEC). *P* values were calculated using a 2-sided Wilcoxon rank-sum test with Bonferroni correction. In the box plots, the central lines, the box limits, and the whiskers represent medians, the interquartile range (IQR), and the minimum/maximum values that are not outliers, respectively. Outliers are shown as points and include any values that are more than 1.5× IQR away from the box.

Previously, using confocal microscopy and scRNA-Seq analysis of dLN cells, we discovered that MARCO^+^ LECs lining the medullary sinuses internalize virus particles and harbor CHIKV RNA at 24 hours after infection (27). Notably, analysis of LECs captured from mock- and CHIKV-infected LNs indicates that the number of floor LECs is reduced in CHIKV-infected compared with uninfected control LNs (27). Floor LECs line the inner layer of the SCS and have intimate contacts with antigen-detecting SCS macrophages (15, 29); this makes them the first LNSCs to encounter cells and foreign antigens entering the LN and suggests that these cells could interact with CHIKV prior to MARCO^+^ LECs in the downstream medullary sinus. CHIKV replication peaks between 24 and 72 hours following infection of WT C57BL/6 mice (6, 7, 11, 27, 32) and can be directly cytopathic (33). Based on these observations, and previous reports that some floor LECs express MARCO (15, 16, 20), we hypothesized that floor LECs interact with CHIKV at earlier times after infection, leading to their reduction by 24 hours after infection. To test this idea, LNSCs from the dLN of mock- and CHIKV-infected mice were profiled by scRNA-Seq at 8 hours after infection. Similar to our previous analysis, single-cell suspensions of LNs were enriched for CD45^–^ stromal cells within the LN by negative selection before processing for scRNA-Seq (Supplemental Figure 1; supplemental material available online with this article; https://doi.org/10.1172/jci.insight.176537DS1) (27). In comparison with our prior analysis of LNSCs at 24 hours (27), fewer viral RNA reads were detected at 8 hours. Thus, we enriched the cDNA libraries for CHIKV RNA using our previously described resampling and resequencing method (34). After enrichment, we clustered cells and identified LN cell types using our established methods (27) (Supplemental Figure 2). Next, we assessed the percentage of reads aligning to the CHIKV genome among total CHIKV- and mouse-specific reads for each individual cell (CHIKV score; Figure 1A). Analysis of cells harboring viral RNA revealed several CHIKV^+^ cell types consisting of different endothelial cell and fibroblast populations (Figure 1B), a finding that is consistent with the known primary tropism of CHIKV for nonhematopoietic cells (8, 35). Among the CHIKV^+^ cell types identified, floor LECs and MARCO^+^ LECs had the highest CHIKV scores and the greatest proportion of CHIKV^+^ cells (Figure 1, C and D), with the floor LEC population containing the highest proportion of CHIKV^+^ cells.

Since CHIKV RNA was predominantly detected in MARCO-expressing LECs at 24 hours after infection (27), we next analyzed *Marco* expression in the CHIKV RNA^+^ and RNA^–^ floor LECs at 8 hours after infection. CHIKV RNA^+^ floor LECs (CHIKV^+^) exhibited greater *Marco* expression than CHIKV RNA^–^ floor LECs (CHIKV^–^) (Figure 1E). Floor and MARCO^+^ LECs are transcriptionally similar but can be distinguished by *Madcam1* expression in floor LECs; *Madcam1* is absent in MARCO^+^ LECs (15). Both CHIKV RNA^+^ and CHIKV RNA^–^ floor LECs exhibited similar *Madcam1* expression, a marker unique to floor LECs (36–38), suggesting that these cells are indeed floor LECs and not misannotated MARCO^+^ LECs (Figure 1F). Collectively, these studies reveal that CHIKV RNA accumulates in 2 subsets of LECs during the first 24 hours of infection and suggest that CHIKV interactions with MARCO are important for viral capture and internalization by endothelial cells in the LN.

### CHIKV RNA^+^ LECs show signs of active CHIKV RNA replication.

At 24 hours, CHIKV RNA-high cells identified by scRNA-Seq had attributes consistent with decreased cell viability and with virus-mediated transcriptional shutoff, including expression of fewer host genes and an increased fraction of reads aligning to mitochondrial genes (27), suggesting that LECs support active CHIKV RNA replication. One marker of active CHIKV RNA replication is the production of a positive-sense subgenomic mRNA that encodes the viral structural polyprotein (39). To provide further evidence of viral RNA replication in LECs, we calculated the ratio (sgRNA ratio) of reads aligning to the viral sgRNA to reads aligning to the full-length viral genome. Consistent with the localization of CHIKV RNA–high cells in our previous study (27), at 24 hours, cells with the highest sgRNA ratio were found predominantly within the MARCO^+^ LEC subset and a cluster of endothelial cells that we were unable to further annotate due to the low number of expressed host genes (unassigned-LEC) (Supplemental Figure 3, A–D). When we further characterized cell types with the highest CHIKV sgRNA ratio at 24 hours, we observed a negative correlation between the sgRNA ratio and the number of mouse genes expressed by MARCO^+^ LECs and unassigned-LECs (Supplemental Figure 3E). In addition, we also identified a positive correlation between the sgRNA ratio and the percentage of mitochondrial reads per cell (Supplemental Figure 3E). Notably, the unassigned-LECs have a higher CHIKV sgRNA ratio, a higher percentage of mitochondrial reads, and a lower number of expressed mouse genes compared with MARCO^+^ LECs, suggesting that these cells could be severely injured MARCO^+^ LECs (Supplemental Figure 3E). To further evaluate LEC viability during CHIKV infection, we used flow cytometry to assess the viability of LECs in the dLN of mock- and CHIKV-infected mice at 1 day after infection, which revealed diminished LEC viability in the dLN of CHIKV-infected mice (Supplemental Figure 3F), consistent with our scRNA-Seq data. Overall, these results show that cells with high levels of viral sgRNA have indications of reduced viability, suggesting that CHIKV RNA replication occurs in LECs that capture and internalize CHIKV particles and leads to cell injury or death.

### LN sinus alteration during pathogenic CHIKV infection.

**Figure 2:**
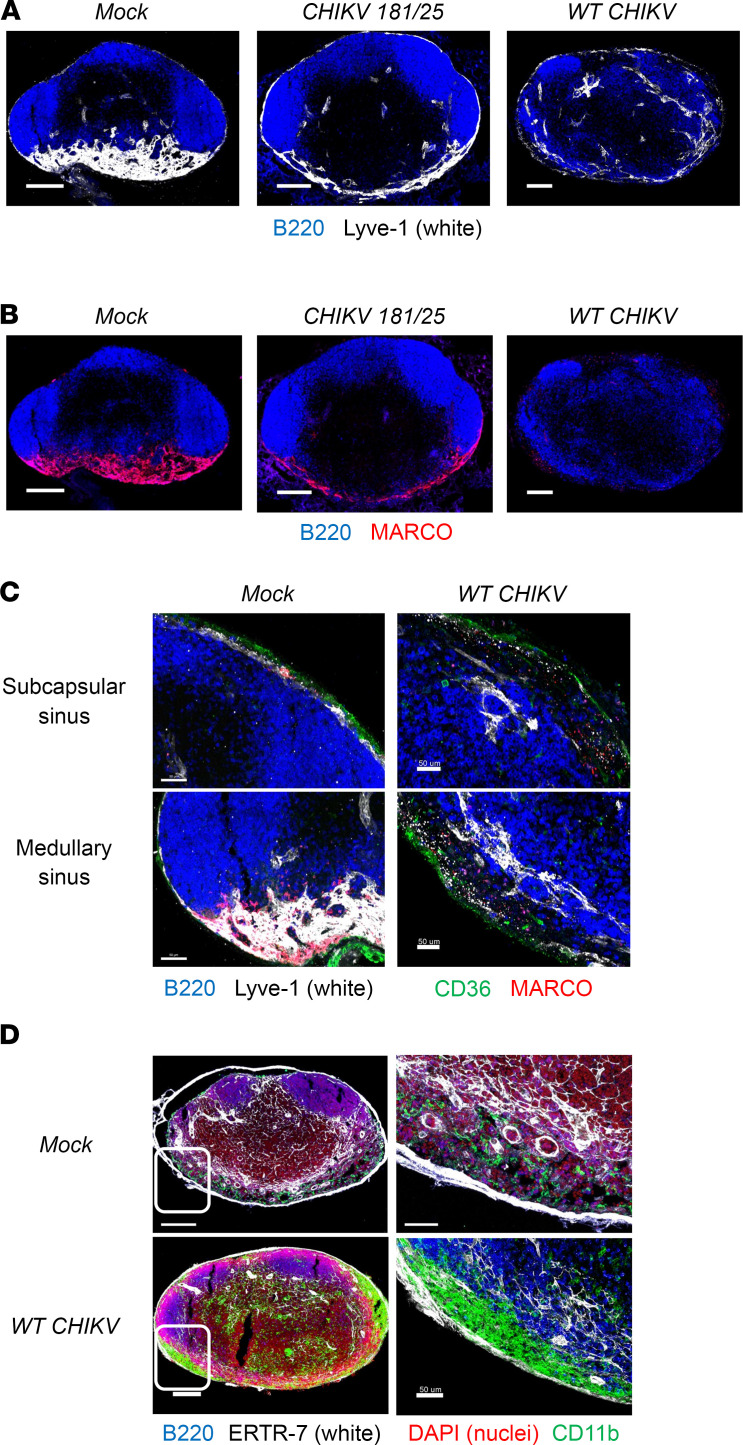
WT CHIKV infection disrupts LEC marker expression and elicits infiltration of LN sinuses. (**A**–**D**) WT mice were mock inoculated (*n* = 3) or inoculated in the footpad with 1 × 10^3^ PFU CHIKV 181/25 (*n* = 5) or WT CHIKV (*n* = 5), and the dLN was collected at 48 hours after infection. (**A**) LN sections stained for B220 (B cells, blue) and Lyve1 (LECs, white). Scale bar: 200 μm. (**B**) LN sections stained for B220 (B cells, blue) and MARCO (red). Scale bar: 200 μm. (**C**) Higher-magnification images of subcapsular and medullary sinus regions in LNs stained for B220 (B cells, blue), Lyve1 (floor and medullary LECs, white), CD36 (ceiling LECs, green), and MARCO (red). Scale bar: 50 μm. (**D**) LNs stained for B220 (B cells, blue), ERTR-7 (fibroblasts, white), nuclei (red), and CD11b (myeloid cells, green). Scale bar: 200 μm (left), 50 μm (right). Images are representative of 3–5 dLNs per group (2 independent experiments).

Our analyses indicate that CHIKV RNA accumulates in multiple subsets of MARCO-expressing LN LECs during infection. To further investigate the fate of these cells, we evaluated Lyve1 and MARCO expression in the dLN during infection with the attenuated CHIKV 181/25 strain, which does not disrupt dLN cellular organization (13), and its parental strain, the pathogenic WT CHIKV AF15561, at 8, 24, and 48 hours after infection using immunofluorescence confocal microscopy. At 8 and 24 hours after infection, Lyve1 and MARCO expression were similar across dLNs from mock-, CHIKV 181/25–, and WT CHIKV–infected mice (Supplemental Figure 4, A and B). Lyve1 signal was observed in both subcapsular and medullary sinus regions, supporting the annotation of both floor and MARCO^+^ LEC subsets in the scRNA-Seq data set from 8 hours after infection and consistent with previous reports supporting the specificity of Lyve1 expression for floor (low level) and MARCO^+^ (high level) LECs (15, 16, 40). MARCO signal was localized predominantly to the LN medullary sinuses, consistent with reports indicating that MARCO expression on LN LECs is regionally distinct (15, 16). However, by 48 hours after infection, Lyve1 signal was greatly reduced and MARCO signal was undetectable in dLNs from WT CHIKV–infected mice (Figure 2, A and B and Supplemental Figure 4C). Higher-magnification imaging of the subcapsular and medullary sinuses of LNs from mock- and WT CHIKV–infected mice using Lyve1 (floor and MARCO^+^ LECs) and CD36 (ceiling LECs) revealed marked expansion of both sinuses in the dLN of mice infected with WT CHIKV (Figure 2C). To investigate the cellular composition of the expanded sinuses, LN sections from mock- and WT CHIKV–infected mice were stained for CD11b, since prior studies identified localization of inflammatory monocytes to the medullary region of the dLN at 24 hours after CHIKV infection (12); the fibroblast marker ERTR-7 to visualize the LN capsule; and DAPI to identify cell nuclei. Indeed, the expanded LN sinuses observed in the dLN of WT CHIKV–infected mice contained numerous CD11b^+^ cells (Figure 2D), supporting the association of inflammatory cellular infiltrates with alteration of resident cells in the LN sinus.

### Pathogenic CHIKV infection alters LN LEC composition.

**Figure 3:**
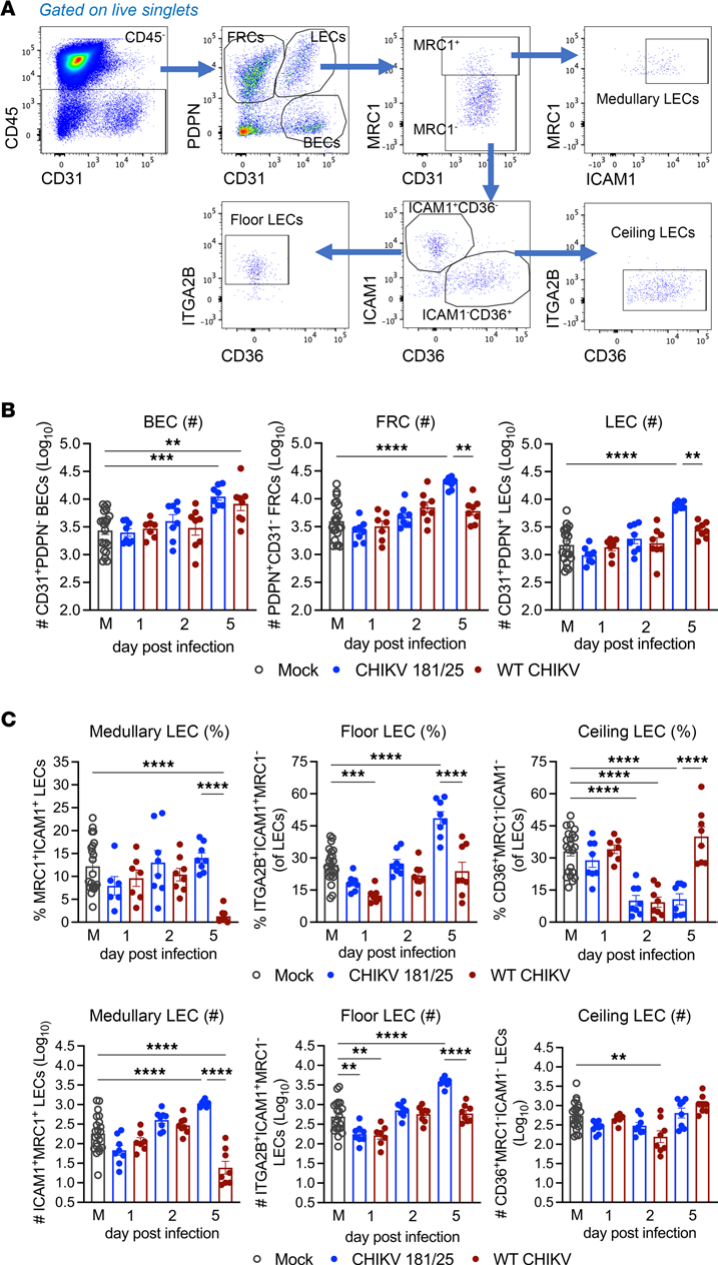
WT CHIKV infection alters LN LEC composition. (**A**–**C**) WT mice were inoculated in the footpad with PBS (*n* = 8) or 1 × 10^3^ PFU CHIKV 181/25 (*n* = 8) or WT CHIKV (*n* = 8) (2 independent experiments). At the time points indicated, the dLN was collected and analyzed by flow cytometry. (**A**) Gating strategy (after gating on viable singlets) to segregate BECs, LECs, and FRCs and further subset LECs into floor, ceiling, and medullary LECs. (**B**) Total number of BECs, FRCs, and LECs. (**C**) Percentage and total number of medullary, floor, and ceiling LECs at each time point. Data presented as mean ± SEM. **P < 0.01; ***P < 0.001; ****P < 0.0001, by 1-way ANOVA with Tukey’s multiple-comparison test.

To evaluate whether loss of Lyve1 and MARCO signal at 48 hours after infection corresponded to the loss of specific LECs or an altered composition of LEC subsets, dLN stromal cells were evaluated at 1, 2, and 5 day after infection by flow cytometry. After excluding nonviable cells, LNSCs were segregated within all CD45^–^ cells based on expression of CD31/PECAM-1 and podoplanin (PDPN) (15, 36, 37): CD31^+^PDPN^–^ BECs, CD31^–^PDPN^+^ FRCs, and CD31^+^PDPN^+^ LECs. LECs subsets were defined using mannose receptor C-type 1 (MRC1), intercellular adhesion molecule 1 (ICAM1), integrin subunit α 2B (ITGA2B), and the scavenger receptor CD36, which have been used successfully in other studies to discriminate medullary (MRC1^+^ICAM1^+^), floor (MRC1^–^ICAM1^+^ITGA2B^+^), and ceiling (MRC1^–^ICAM1^–^CD36^+^) LEC subsets (36, 41) (Figure 3A). The total number of BECs, FRCs, and LECs was similar between mock-, CHIKV 181/25–, and WT CHIKV–infected LNs at 1 and 2 days after infection (Figure 3B). At 5 days after infection, while the number of BECs increased to a similar extent following infection with either CHIKV 181/25 or WT CHIKV (Figure 3B), FRC and LEC numbers increased solely in CHIKV 181/25–infected LNs (Figure 3B), suggesting that WT CHIKV infection alters the proliferation or survival of these LNSC subtypes. Further segregation of LECs into medullary, floor, and ceiling subtypes revealed that both the percentage and number of medullary LECs was reduced during WT CHIKV infection compared with CHIKV 181/25 infection, whereas the number of ceiling LECs was unchanged at 5 days after infection (Figure 3C). The percentage and number of floor LECs was also reduced during WT CHIKV infection compared with CHIKV 181/25 infection (Figure 3C). Notably, there was a small but significant reduction in the percentage and number of dLN floor LECs between mock and WT CHIKV–infected mice at 1 day after infection, suggesting that these cells, which interact with the virus early after infection, could be damaged as a result. These data suggest that WT CHIKV infection impairs the expansion and/or maintenance of specific regional LEC subsets. Notably, medullary and floor LECs include all the MARCO-expressing LECs in the LN, which are LN cell types predominantly targeted by CHIKV early after infection (Figure 1, B–D) (27), suggesting that these changes could be due to CHIKV-MARCO interactions.

### Alteration of LN LEC composition is dependent on CHIKV-MARCO interactions.

**Figure 4:**
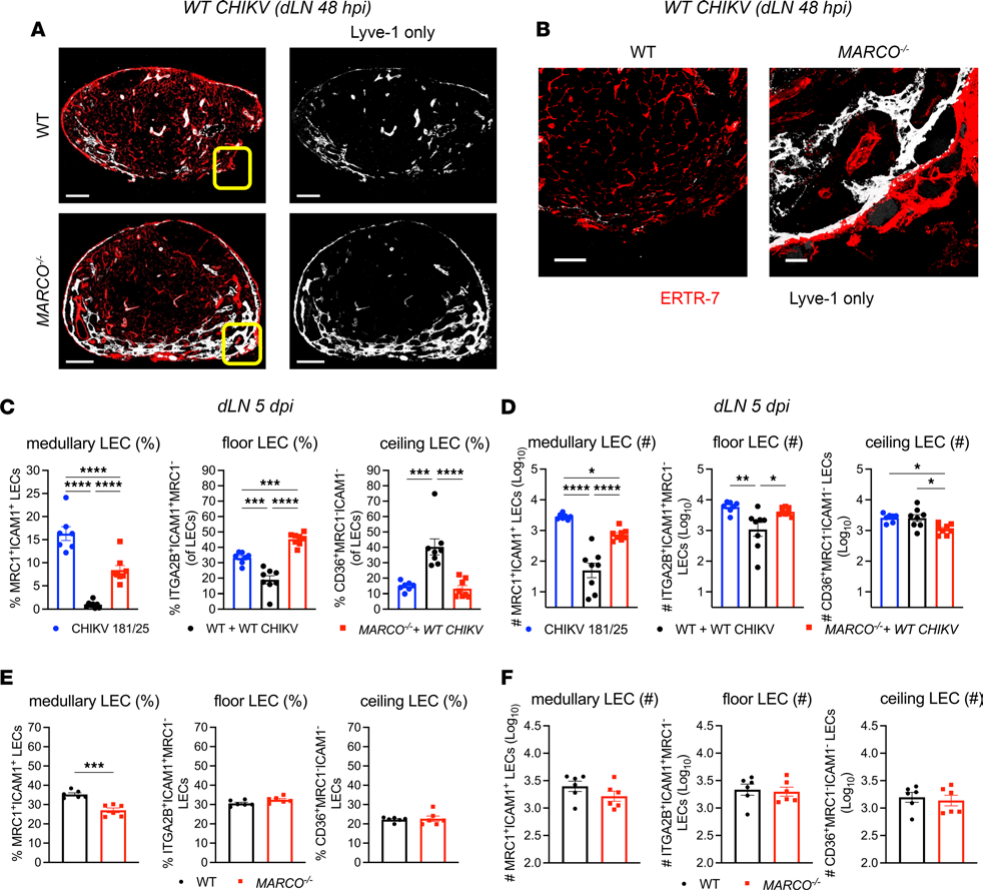
Loss of Lyve1 and changes in LEC subset composition are MARCO dependent. (**A** and **B**) WT and *MARCO^–/–^* mice were inoculated in the footpad with 1 × 10^3^ PFU WT CHIKV (*n* = 7–8). At the indicated time points, the dLN was collected for analysis by immunofluorescence confocal microscopy or flow cytometry. (**A**) LN sections were stained for Lyve1 (LECs, white) and ERTR-7 (fibroblasts, red). Scale bar: 200 μm. (**B**) Higher-magnification images of the indicated LN sinus region (yellow box). Scale bar: 50 μm. Images are representative of 5 dLNs per group (2 independent experiments). (**C** and **D**) Percentage and total number of medullary, floor, and ceiling LECs at 5 days after infection. (**E** and **F**) Percentage and total number of medullary, floor, and ceiling LECs. Data are combined from 2 independent experiments and presented as mean± SEM. *P < 0.05; **P < 0.01; ***P < 0.001; ****P < 0.0001, by 1-way ANOVA with Tukey’s multiple-comparison test (**C** and **D**) or Student’s *t* test (**E** and **F**).

Our prior studies identified a role for the scavenger receptor MARCO in early viral accumulation in the dLN and restricting early viral dissemination to distal tissues (27, 42). Furthermore, our data indicate that CHIKV RNA accumulates predominantly in subsets of LECs that express MARCO. Thus, we hypothesized that CHIKV-MARCO interactions promote LN sinus alteration and altered LEC subset composition in the dLN of WT CHIKV–infected mice. To assess this, dLNs were evaluated at 48 hours after infection by confocal microscopy following CHIKV infection of WT and *MARCO^–/–^* mice. In contrast to the sparse Lyve1 signal in the dLN of WT mice, the dLN of CHIKV-infected *MARCO^–/–^* mice exhibited more robust Lyve1 expression (Figure 4A). Higher-magnification imaging of the medullary sinus highlights the difference in Lyve1 expression in the dLN, where Lyve1 signal is substantially diminished in WT mice, while *MARCO^–/–^* mice maintain robust Lyve1 expression (Figure 4B). These data suggest that MARCO promotes the loss of Lyve1 expression or LECs in the dLN during CHIKV infection. Furthermore, analysis of LN LEC subsets at 5 days after infection reveals that, in WT CHIKV–infected *MARCO^–/–^* mice, medullary and floor LEC populations were increased compared with WT CHIKV–infected WT mice (Figure 4, C and D), indicating that the altered composition of LECs during CHIKV infection was MARCO dependent. In addition, the percentage of ceiling LECs was similar in WT CHIKV–infected *MARCO^–/–^* mice to CHIKV 181/25–infected WT mice in contrast to the higher percentage of ceiling LECs in WT CHIKV–infected WT mice (Figure 4C), suggesting that the change in the proportion of ceiling LECs may be a component of the LNSC response to CHIKV infection. Importantly, the composition of LN LEC subsets was similar in naive WT and *MARCO^–/–^* mice (Figure 4, E and F), supporting the conclusion that disruption of LN LEC composition during CHIKV infection was MARCO dependent. Overall, these data demonstrate a role for MARCO in the alteration of specific LN LEC populations during pathogenic WT CHIKV infection.

### Inflammatory gene expression in LNSCs during CHIKV infection.

**Figure 5:**
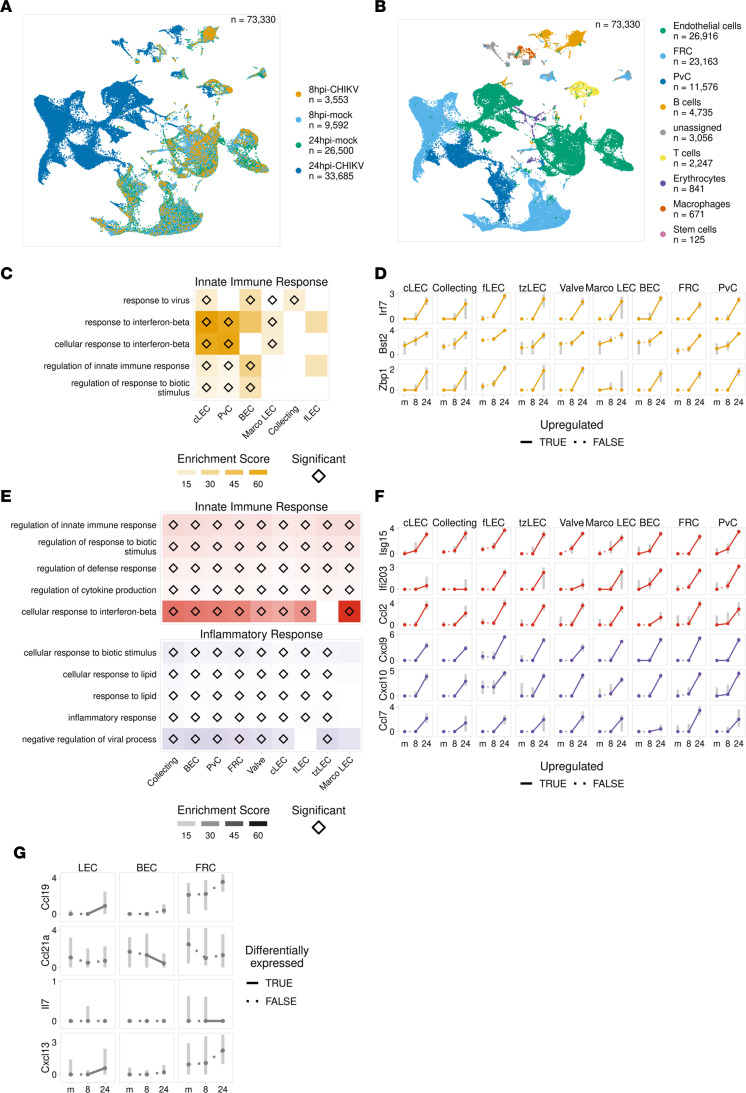
LNSCs exhibit a dominant proinflammatory response 8 hours after CHIKV infection. (**A**–**G**) Cells from the dLN were collected from mock- or WT CHIKV-inoculated mice at 8 or 24 hours after infection. Cells were subjected to CD45^+^ cell depletion and then scRNA-Seq. (**A**) UMAP showing all cells from the 8- and 24-hour time points colored by sample. (**B**) UMAP showing all cells from the 8- and 24-hour time points colored by cell type. (**C**) Enrichment scores for each cell type for the top 5 terms from the primary gene ontology module identified for the 8-hour time point. Enrichment score is the fraction of upregulated genes overlapping the term divided by the fraction of background genes overlapping the term. Significantly enriched GO terms are marked by a diamond. (**D**) A selection of top upregulated genes for terms significantly enriched at 8 hours. Points show the median expression for mock (m), 8 hours, and 24 hours samples; gray bars show the interquartile range. A solid line indicates the gene is significantly upregulated between the time points. (**E**) Enrichment scores for the primary GO modules for the 24-hour time point, as described in **C**. (**F**) A selection of top upregulated genes for the terms identified at 24 hours, plotted as described in **D**. (**G**) Expression of select homeostatic chemokines among the major LNSC types, plotted as described in **D**. A solid line indicates that the gene is differentially expressed between the time points.

We next evaluated differential gene expression in LNSCs from mock- and WT CHIKV–infected mice at 8 and 24 hours after infection using our scRNA-Seq data sets. Uniform manifold approximation and projection (UMAP) of all LNSCs from mock- and WT CHIKV–infected mice at both 8 and 24 hours after infection revealed that LNSCs from WT CHIKV–infected mice at 8 hours clustered strongly with LNSCs from mock-infected mice, whereas LNSCs from WT CHIKV–infected mice at 24 hours were strongly segregated from the other populations (Figure 5, A and B) and this was consistent when coloring the UMAP by cell type (Figure 5B). We next identified gene ontology (GO) terms (biological process) for genes upregulated in each cell type. To identify the predominant gene expression programs upregulated during the first 24 hours of CHIKV infection, we clustered GO terms for each time point into distinct modules based on similarity. From this analysis, the primary gene expression module upregulated at 8 hours after infection consists of factors associated with the innate immune response (Figure 5C), including *Bst2*, which is broadly upregulated in most cell types at 8 hours and can promote retention of CHIKV particles at the host cell membrane to prevent virus release (43, 44). We also detected upregulation of *Zbp1*, a key factor in sensing cytosolic DNA during virus-induced cell damage, and *Irf7*, another key factor in the induction of antiviral cytokines such as IFN-β (45, 46) (Figure 5D). When we compared changes in gene expression between the 24- and 8-hour time points, we identified a similar innate immune response module and observed further upregulation of *Bst2*, *Zbp1*, and *Irf7* (Figure 5, D and E). By 24 hours after infection, we also detected increased expression of genes involved in a broader inflammatory response, including *Ccl2*, *Cxcl9*, *Cxcl10*, and *Ccl7*, which were upregulated across the major LNSC subsets (Figure 5, E and F). However, minor differences in the degree of expression of specific genes were noted, with *Ccl7* upregulated to a lower degree in MARCO^+^ LECs and perivascular cells (PvCs) than FRCs, consistent with fibroblasts being a primary CCL7-producing stromal cell type (Figure 5F) (47, 48). In addition, CCL2 is a potent chemoattractant for monocytes, which previous studies demonstrated are detrimental to LN structure and function (12, 49); the high expression of *Ccl2* detected in LECs suggests that these cells may contribute to early recruitment of inflammatory monocytes. We also evaluated expression of important LNSC homeostatic chemokines including *Ccl21a*, *Il7*, *Cxcl13*, and *Ccl19* (Figure 5G). The expression level of these genes was largely unchanged or diminished in LNSCs of WT CHIKV–infected mice when compared with mock-infected mice (Figure 5G), indicating that the primary effect of CHIKV infection on the transcriptome of LNSCs is activation of antiviral and inflammatory gene expression programs.

### MARCO expression triggers a rapid LN inflammatory response.

**Figure 6:**
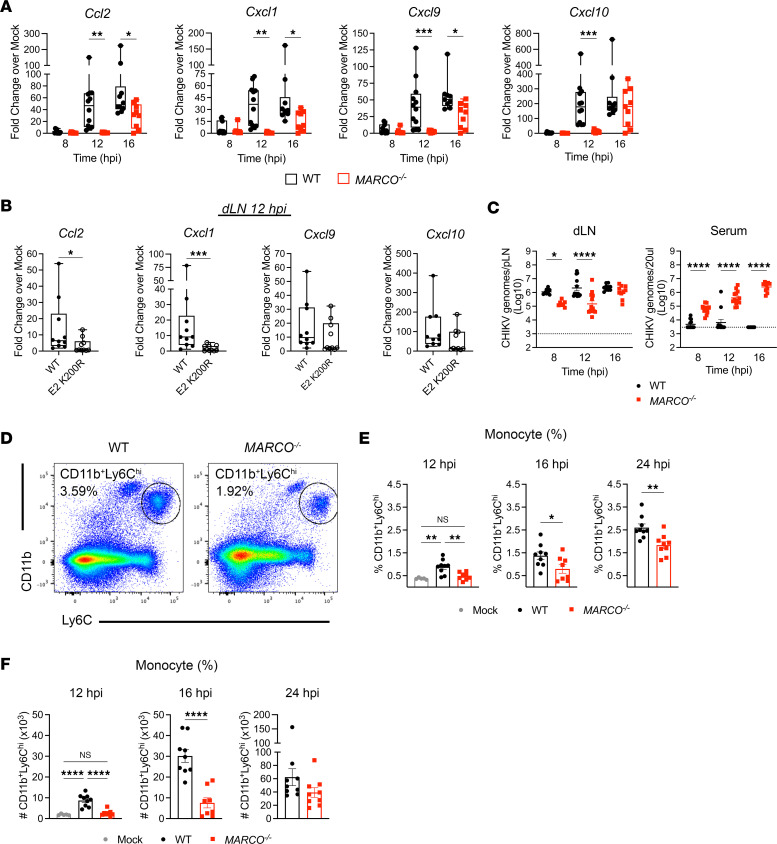
CHIKV-MARCO interactions promote LN inflammation. (**A**–**E**) WT and *MARCO^–/–^* mice were inoculated in the footpad with 1 × 10^3^ PFU WT CHIKV (*n* = 9–13) or CHIKV^E2^ ^K200R^ (*n* = 10) (2–3 independent experiments). At the indicated time points, the dLN was collected for gene expression and viral RNA analysis by qPCR (**A**–**C**) or for inflammatory myeloid cells by flow cytometry (**D**–**F**). (**A**) Expression of chemokines at 8, 12, and 16 hours after infection. (**B**) Expression of chemokines at 12 hours after infection. (**C**) CHIKV RNA in the dLN and serum. (**D**) Representative flow cytometry plots of inflammatory CD11b^+^Ly6C^hi^ monocytes. (**E**) Percentage of monocytes in the dLN at 12, 16, and 24 hours after infection. (**F**) Total number of monocytes per dLN at 12, 16, and 24 hours after infection. *P <0.05; **P < 0.01; ***P < 0.001; ****P < 0.0001, by 1-way ANOVA with Tukey’s multiple-comparison test (**A** and **C**) or Student’s *t* test (**B**, **E**, and **F**).

The presence of inflammatory CD11b^+^ cells in the expanded sinuses of WT CHIKV–infected LNs (Figure 2, B and C), retention of Lyve1 signal in *MARCO^–/–^* mice (Figure 4A), and high expression of monocyte chemoattractant *Ccl2* in LNSCs at 24 hours after infection (Figure 5F) suggest that CHIKV-MARCO interactions promote LN inflammation via regulation of inflammatory chemokine expression and recruitment of inflammatory monocytes. To investigate this hypothesis, inflammatory chemokine mRNA expression (*Ccl2*, *Cxcl1*, *Cxcl9*, and *Cxcl10*) was assessed in whole LNs from WT and *MARCO^–/–^* mice at time points (8, 12, and 16 hours after infection) prior to the dominant type I IFN response observed at 24 hours (Figure 5) (12). Consistent with our scRNA-Seq analysis, little to no upregulation of these chemokines was observed in WT CHIKV–infected WT or *MARCO^–/–^* mice at 8 hours after infection. However, by 12 hours after infection, the expression of *Ccl2*, *Cxcl1*, *Cxcl9*, and *Cxcl10* in the dLN of WT CHIKV–infected WT mice, but not *MARCO^–/–^* mice, was significantly increased in comparison with mock-infected mice (Figure 6A). By 16 hours after infection, chemokine expression was increased in both WT CHIKV–infected WT and *MARCO^–/–^* mice in comparison with mock-infected mice; however, *Ccl2*, *Cxcl1*, and *Cxcl9* expression remained significantly higher in the dLN of WT mice (Figure 6A). To further address whether direct CHIKV-MARCO interactions and subsequent viral internalization promote early inflammatory chemokine expression in the dLN, chemokine expression in the dLN was assessed at 12 hours after infection in WT mice infected with WT CHIKV or CHIKV^E2^ ^K200R^, which lacks interaction with MARCO (27, 42, 50). The expression of *Ccl2* and *Cxcl1* was significantly increased in the dLN of WT CHIKV–infected mice compared with CHIKV^E2^ ^K200R^–infected mice (Figure 6B), whereas differences in *Cxcl9* and *Cxcl10* expression were not statistically significant. Concurrent with the lower inflammatory chemokine expression in *MARCO^–/–^* mice, we detected less CHIKV RNA in the dLN of *MARCO^–/–^* mice at 8 and 12 hours after infection (Figure 6C). These data suggest that CHIKV-MARCO interactions promote inflammatory gene expression in the dLN.

To determine if MARCO also promotes infiltration of the LN with CD11b^+^ cells during CHIKV infection, we evaluated accumulation of inflammatory monocytes in the dLN of WT and *MARCO^–/–^* mice using flow cytometry (Figure 6D). Consistent with higher *Ccl2* expression in WT mice at 12 and 16 hours after infection, the percentage (Figure 6E) and total number (Figure 6F) of inflammatory Ly6C^hi^ monocytes (CD45^+^CD11c^–^CD11b^+^Ly6C^hi^Ly6G^–^) in the dLN was significantly greater in WT mice at 12 and 16 hours after infection compared with *MARCO^–/–^* mice. By 24 hours after infection, the percentage of inflammatory monocytes remained significantly higher in WT mice than in *MARCO^–/–^* mice (Figure 6E), although the difference in monocyte numbers was not statistically significant (Figure 6F). These data suggest that CHIKV-MARCO interaction induces a rapid early proinflammatory response, recruiting pathogenic monocytes that cause disruption of dLN cellular organization and impair B cell responses (12).

### Pathogenic CHIKV infection impairs foreign antigen acquisition by LECs.

**Figure 7:**
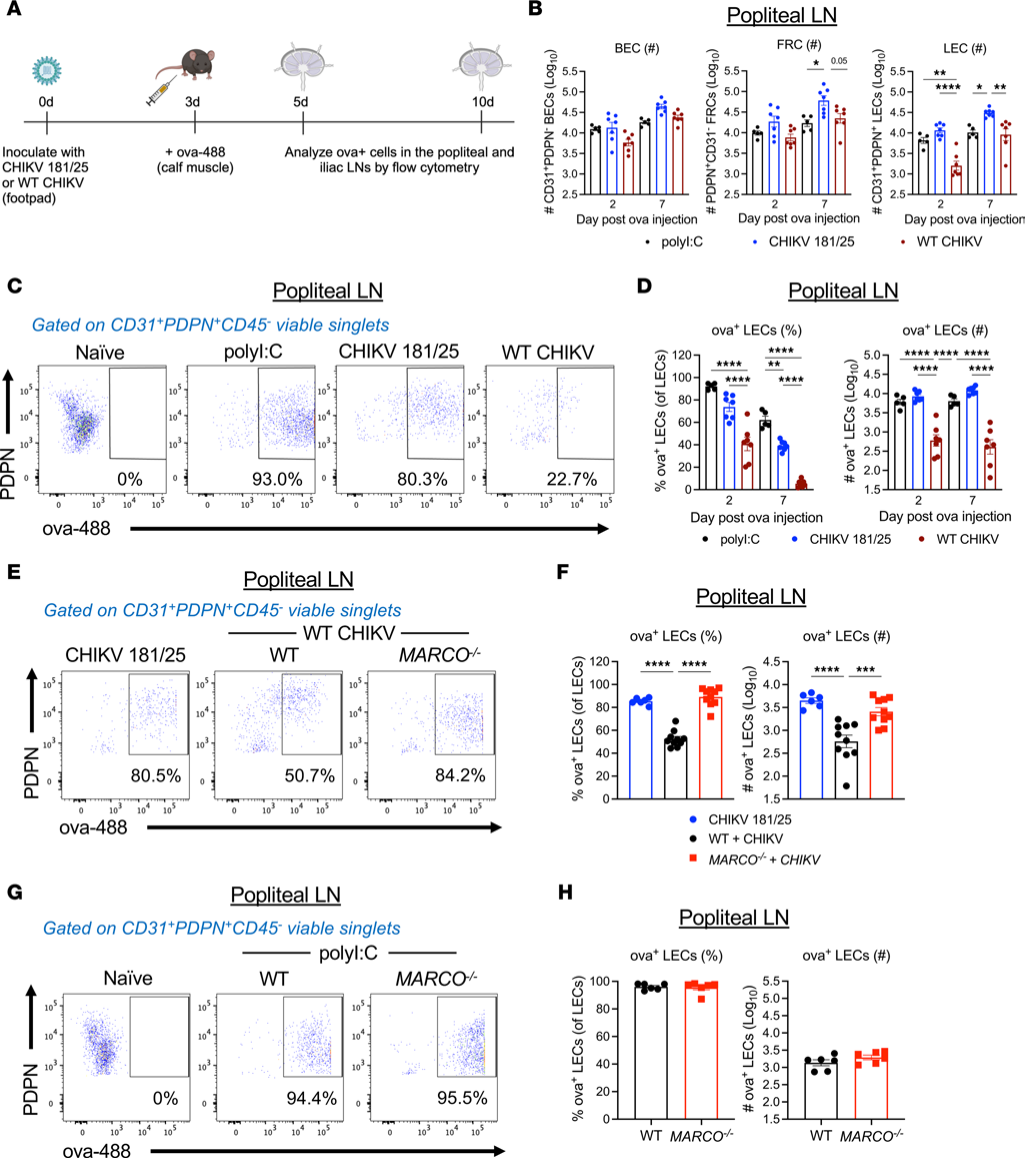
WT CHIKV infection impairs antigen acquisition by LECs in a MARCO-dependent manner. (**A**) WT and *MARCO^–/–^* mice were mock inoculated (*n* = 5) or inoculated in the footpad with 1 × 10^3^ PFU CHIKV 181/25 (*n* = 8) or WT CHIKV (*n* = 8) (2 independent experiments). At 72 hours after infection, mice were inoculated with 10 μg ova-488 in both calf muscles (20 μg total). As controls, naive WT and *MARCO^–/–^* mice were injected with 10 μg ova-488 and 5 μg polyI:C. Ova^+^ LECs in the popliteal LN were enumerated by flow cytometry at the indicated time points. (**B**) LNSC numbers in the popliteal LN following ova immunization. (**C**) Representative flow cytometry plots showing ova^+^ LECs. (**D**) The percentage and total number of ova^+^ LECs. (**E**) Representative flow cytometry plots showing ova^+^ LECs. (**F**) Percentage and number of ova^+^ LECs. (**G**) Representative flow cytometry plots showing ova^+^ LECs in mock-infected WT and *MARCO^–/–^* mice inoculated with polyI:C/ova-488. (**H**) Percentage and number of ova^+^ LECs. *P <0.05; **P < 0.01; ***P < 0.001; ****P < 0.0001, by 1-way ANOVA with Tukey’s multiple-comparison test (**B**, **D**, and **F**) or Student’s *t* test (**H**).

A key function of LN LECs is the ability to acquire and retain foreign antigen to promote long-lived adaptive immunity following both viral infection and vaccination (21, 24–26). Prior studies showed that fluorescently labeled ovalbumin (ova) or influenza nucleoprotein (NP) is specifically acquired by LN LECs upon s.c. injection of mice experiencing an active viral infection or when delivered with an adjuvant, such as polyI:C (24–26). To evaluate the functional capacity of LN LECs to acquire antigen during CHIKV infection, we immunized CHIKV 181/25– or WT CHIKV–infected mice with 10 μg ova-488 in both calf muscles (20 μg/mouse total) at 3 days after infection, at which point the dLN of WT CHIKV–infected mice is substantially disorganized (13), and assessed the proportion of ova^+^ LNSCs 2 and 7 days later (Figure 7A). As a positive control, we immunized naive mice in both calf muscles with 10 μg ova-488 and 5 μg polyI:C per calf (24, 25). Consistent with prior studies (24, 25), ova acquisition was highly specific to LN LECs, as little to no ova was detected in BECs or FRCs (Supplemental Figure 5, A and B), confirming that we could measure LEC antigen acquisition by this method (25). We evaluated ova^+^ LNSCs in both the popliteal (first footpad dLN) and iliac (next dLN in sequence) LNs (51) to determine if any differences in ova acquisition were associated with impaired lymphatic drainage. Comparison of LNSC populations in the popliteal LN of ova-immunized/WT CHIKV–infected mice revealed that the number of LECs in WT CHIKV–infected mice was diminished at both 2 and 7 days after ova injection in the popliteal LN compared with CHIKV 181/25–infected mice (Figure 7B). FRC and BEC numbers were not significantly different between WT CHIKV–infected mice and CHIKV 181/25–infected mice at both 2 and 7 days after ova immunization in the popliteal LN (Figure 7B), suggesting that the adverse effects of WT CHIKV are specific to LECs. By gating on ova^+^ LECs in naive mice as a negative control, we found that both the percentage and number of ova^+^ LECs was reduced during WT CHIKV infection compared with CHIKV 181/25 infection in the popliteal LN (Figure 7, C and D). The difference in ova^+^ LEC number was greater at both 2 and 7 days after ova in the popliteal LN between CHIKV 181/25 and WT CHIKV infection compared with total LEC number alone, suggesting that differences in ova acquisition within the popliteal LN cannot be attributed solely to differences in total LEC numbers. Notably, similar findings were observed in the iliac LN (Supplemental Figure 5, C–E), although the magnitude of the effects was reduced compared with the popliteal LN, suggesting that the detrimental effects of WT CHIKV infection on LN structure and function are reduced as cells and antigen move farther downstream in the lymphatic drainage network. Consistent with the role of MARCO in alteration of LN LEC composition and enhanced inflammation during WT CHIKV infection, we observed robust antigen acquisition by LECs in WT CHIKV–infected *MARCO^–/–^* mice, similar to that observed in CHIKV 181/25–infected WT mice (Figure 7, E and F), indicating that MARCO promotes the disruption of both LEC composition and function during CHIKV infection. Importantly, antigen acquisition by LECs in uninfected mice was MARCO independent (Figure 7, G and H), further indicating that CHIKV infection impairs LEC function via MARCO. Overall, these data suggest that pathogenic CHIKV infection impairs the ability of LECs to acquire antigen upon a secondary foreign challenge, and this impairment could have implications for the strength and success of downstream adaptive immunity to respond to that challenge.

## Discussion

Previous work demonstrates that LN cellular organization and immune responses are disrupted during CHIKV infection (12, 13); however, the specific virus-host interactions that promote these aberrant LN responses remained unknown. Recently, we found that CHIKV particles are internalized by MARCO^+^ medullary LECs in the LN (27). Building on these findings, here we show that CHIKV RNA accumulates within floor and medullary LN LECs, that this accumulation is associated with expression of the scavenger receptor MARCO by these cells, and that these cells may support active viral RNA replication. Moreover, CHIKV infection was associated with the rapid induction of an antiviral and inflammatory gene expression program across LNSCs that was accelerated by CHIKV-MARCO interactions. In addition, we found that, as CHIKV infection progressed, both floor and medullary LECs diminished in number in a MARCO-dependent manner. This unique viral targeting and/or capture by LN LECs raised questions about how virus interactions with LNSCs influence the function of these cells in immunity. Indeed, we found that acquisition of vaccine antigen by LECs was reduced following pathogenic CHIKV infection in a MARCO-dependent manner, suggesting that virus-LNSC interactions can influence subsequent secondary responses.

Both previous work and our data here indicate that CHIKV RNA accumulates within specific subsets of LECs in the dLN (27); CHIKV RNA preferentially accumulated in LECs expressing higher levels of the scavenger receptor MARCO (27), emphasizing the critical role of MARCO in facilitating CHIKV targeting of LECs, consistent with our recent report that MARCO can facilitate viral internalization in vitro (50). The accumulation of CHIKV RNA in MARCO^+^ floor LECs and medullary MARCO^+^ LECs also reflects the transit over time of viral particles through the LN sinuses and prompted the question of whether these cells are permissive to viral replication. In fact, we identified an increased ratio of CHIKV sgRNA within MARCO^+^ LECs at 24 hours after infection and both a lower number of expressed host genes and a higher percentage of mitochondrial reads in those cells, suggesting active viral RNA replication. These data contrast with a prior report indicating that LNSCs are not targets of CHIKV infection (13). One explanation could be that there is some viral RNA replication within LECs but the infection is ultimately abortive due to little to no virion production and release. Indeed, expression of the antiviral factor *Bst2*/tetherin was increased at both 8 and 24 hours after infection in LECs, and we observed robust expression of type I IFN–stimulated genes at 24 hours after infection, which together could limit virus replication. Alternatively, due to the small population of LECs that interact with CHIKV, CHIKV^+^ cells may be difficult to detect by methods less sensitive than RNA-Seq. Although CHIKV can interact with a multitude of cell surface proteins for attachment on many cell types, including endothelial cells, other studies have predominantly identified fibroblasts, skeletal muscle cells, and macrophages as targets for active CHIKV replication (8, 35, 52, 53). Indeed, signs of active viral RNA replication in LNSCs are unique and have rarely been identified. Instead, much research has focused on LN resident macrophages, which interact with and can be targeted by viruses such as Zika virus, vaccinia virus, and vesicular stomatitis virus (54–56). However, lymphocytic choriomeningitis virus (LCMV) targets murine FRCs within lymphoid organs, and importantly, LCMV infection of FRCs was higher for the clone 13 strain, which establishes a persistent infection, than the Armstrong strain, which is cleared efficiently by CD8^+^ T cells (57). In contrast, while multiple studies have shown that FDCs can trap HIV-1 and maintain infectious virions, representing a potential viral reservoir during chronic infection, FDCs are not permissive to HIV-1 replication; thus, viral accumulation in FDCs appears to be an unintended consequence rather than the result of viral targeting of FDCs (58, 59). While more investigation is needed, viral targeting of LNSCs by CHIKV and LCMV, 2 viruses associated with chronic disease, suggests that viral targeting of LNSCs may have a critical role in the establishment of chronic viral infection.

A key marker of the floor and medullary LEC populations that harbor CHIKV is Lyve1, and this distinguishes them from other LEC subsets (15, 16, 20, 26, 40). LNs from WT CHIKV but not attenuated CHIKV 181/25–infected mice displayed a reduced and spatially altered expression of Lyve1 by 48 hours after infection as well as a loss of MARCO expression, suggesting that WT CHIKV infection disrupts Lyve1^+^ LECs. In lymphatic vessels, Lyve1 expression is negatively regulated by inflammatory cytokines (60). Notably, WT CHIKV infection of *MARCO^–/–^* mice did not disrupt Lyve1 expression, suggesting that the perturbations of Lyve1 expression observed in WT mice could be driven by MARCO-promoted inflammatory responses. Consistent with this idea, we found that the presence of MARCO accelerated and promoted inflammatory gene expression in the LN. Diminished inflammatory gene expression in *MARCO^–/–^* mice was associated with reduced viral RNA accumulation in the dLN, suggesting that MARCO may accelerate inflammation by facilitating viral RNA accumulation in LN LECs. Furthermore, we observed numerous CD11b^+^ cells within the disrupted LN sinuses at 48 hours after infection, and the presence of MARCO contributed to the accumulation of inflammatory monocytes in the dLN.

Despite the altered expression and spatial distribution of Lyve1 expression observed by confocal microscopy, total LEC, medullary, and floor LEC numbers remained similar in LNs from mock-, CHIKV 181/25–, and WT CHIKV–infected mice at 48 hours after infection when evaluated by flow cytometry. However, by 5 days after infection, we detected decreased numbers of medullary and floor LECs in LNs from WT CHIKV–infected mice compared with those from mice infected with CHIKV 181/25, which induces less LN inflammation and does not disrupt LN cellular organization (12, 13). These changes were specific to floor and medullary LECs, the LEC subsets that harbor CHIKV RNA based on scRNA-Seq. Notably, both floor and medullary LEC numbers were restored in LNs from WT CHIKV–infected *MARCO^–/–^* mice, suggesting that these changes are a consequence of CHIKV targeting of MARCO-expressing LECs. Unexpectedly, we observed a reduced proportion of ceiling LECs among total LECs during CHIKV 181/25 infection compared with WT CHIKV at 5 days after infection, yet total ceiling LEC numbers were similar. It is possible that both *Cd36* and *Icam1* expression were altered in ceiling LECs by cytokines and chemokines expressed in the dLN in the first 5 days after infection, biasing our gating analysis. Furthermore, we also observed a reduced proportion of ceiling LECs in WT CHIKV–infected *MARCO^–/–^* mice similar to that seen in CHIKV 181/25–infected WT mice, supporting the idea that this change is part of a functional LN immune response. One caveat to comparison of our flow cytometry and confocal microscopy data is that the major LEC subsets were identified using different surface proteins. Thus, there could be microenvironment-dependent changes in expression of key markers of LEC subsets that complicate interpretation of the cell populations identified by flow cytometry, such as upregulation of ITGA2B on LECs in response to inflammation (37, 61). Notably, comparison of LEC subsets during homeostatic and response-to-stimuli conditions using scRNA-Seq suggests that floor LECs undergo the greatest transcriptional alteration following stimulus with the TLR7 agonist imiquimod, with moderate alteration observed in medullary LECs and the least changes observed in ceiling LECs (40). However, we note that total LEC numbers in the dLN, as determined by CD31 and PDPN, were reduced during WT CHIKV infection compared with CHIKV 181/25 infection, suggesting that the major changes in LEC subsets are accurate. A previous report investigating the mechanisms dictating expansion and contraction of LN LECs during an immune response demonstrated that type I IFN and PD-L1 both inhibit early LEC division and that a decrease in PD-L1 was sufficient to increase LEC proliferation (62). Given the early inflammatory response in the dLN that occurs during WT CHIKV infection, signaling by type I IFN–stimulated genes, such as PD-L1, may prevent expansion of LN floor LECs.

LEC functions include maintenance of LN homeostatic chemokine gradients, acquisition, and storage of antigen to promote memory CD8^+^ T cell responses through antigen exchange with migratory DCs and maintenance of peripheral self-tolerance (24-26, 28, 63, 64). We found that the capacity of LN LECs to acquire antigen after a secondary immunization was specifically impaired during WT CHIKV infection. In contrast, LEC antigen acquisition in mice infected with CHIKV 181/25 was similar to that observed in naive mice stimulated with polyI:C, consistent with data showing that viral infection can also induce LEC antigen acquisition (24). Antigen acquisition was markedly reduced 2 days after immunization in WT CHIKV–infected mice in a MARCO-dependent manner, and retention of that antigen also may be impaired as the number of ova^+^ LECs remained constant between 2 and 7 days after immunization in CHIKV 181/25–infected mice but decreased in WT CHIKV–infected mice. Overall, these data provide evidence that LNSC-targeting viruses that disrupt the function of the cells represent a challenge for vaccination campaigns, since patients recently infected with such a virus may need to delay immunization to generate stronger, more protective vaccine-specific responses. Importantly, while CHIKV induces both LEC alteration and dysfunction in a MARCO-dependent manner, MARCO itself is not required for optimal LEC function, since LECs from uninfected WT and *MARCO^–/–^* mice stimulated with polyI:C acquired antigen equally well and the composition of LN LEC subsets was also similar in naive WT and *MARCO^–/–^* mice.

In summary, our work demonstrates that CHIKV interactions with specific subsets of LECs expressing the scavenger receptor MARCO is associated with remodeling of the LNSC transcriptome, extensive LN inflammation, and dysfunction of LN LECs. These findings suggest that CHIKV-LEC interactions contribute to impaired downstream LN function and impaired adaptive immunity during CHIKV infection.

## Methods

### Cells.

BHK-21 cells (ATCC CCL-10) were grown at 37°C and 5% CO_2_ in α-minimal essential media (Invitrogen) supplemented with 10% FBS (HyClone), 10% tryptose phosphate broth, penicillin and streptomycin, and 0.29 mg/mL L-glutamine (all Gibco).

### Viruses.

CHIKV AF15561, AF15561^E2^ ^K200R^, and 181/25 were generated as previously described (65). Briefly, plasmids were linearized by NotI (NEB) digestion and used as a template for in vitro transcription with SP6 DNA-dependent RNA polymerase (Ambion). RNA transcripts were electroporated into BHK-21 cells, and 24 hours later, cell culture supernatant was collected and clarified by centrifugation (1,721*g*) for 20 minutes at 4°C, aliquoted, and stored at –80°C. Viral titers were determined by plaque assay or by quantification of RNase-resistant viral genomes by quantitative PCR (qPCR) as previously described (7, 13).

### Mouse experiments.

Mice were bred in specific pathogen–free facilities at the University of Colorado Anschutz Medical Campus. All mouse studies were performed in an animal biosafety level 3 laboratory. WT C57BL/6J (stock no. 000664) mice were acquired from The Jackson Laboratory, and congenic *MARCO^–/–^* mice were provided by Dawn Bowdish (McMaster University, Hamilton, Ontario, Canada) (66). Mice were anesthetized with isoflurane vapors and inoculated with the indicated dose of virus in a 10 μL volume via s.c. injection into the rear footpad. For scRNA-Seq and flow cytometry experiments, mice were inoculated with an equal dose of virus in both rear footpads; for microscopy, mice were inoculated in a single footpad. Since sex-based difference have not been observed in the CHIKV infection model, WT male mice were purchased commercially and were age matched and distributed randomly across groups. Based on prior studies of LN inflammation during CHIKV infection, mice 4 weeks of age were used in all experiments (12, 13). Experimental animals were humanely euthanized at defined endpoints by exposure to isoflurane vapors followed by bilateral thoracotomy.

### Preparation of single-cell suspensions for single-cell mRNA sequencing.

LNs were pooled into individual replicates (3 replicates; LNs from 5 mice pooled per replicate) and mechanically homogenized using a 22G needle in Click’s medium (Sigma-Aldrich) supplemented with 5 mg/mL liberase DL (Roche, 05401160001) and 2.5 mg/mL DNase (Roche, 10104159001) and incubated for 1 hour at 37°C. Cell suspensions were enriched for CD45^–^ cells by labeling cells with PE-conjugated anti-mouse CD45 (30-F11), CD140A (APA5), and Ter119 (Ter119) monoclonal antibodies monoclonal antibodies (all from BioLegend) and subsequent depletion of PE-labeled cells using Miltenyi anti-PE microbeads (catalog 130-048-801) and MACS LS (catalog 130-042-401) columns according to the manufacturer’s instructions with the following modifications: (a) we used 25% of the recommended volume of anti-PE microbeads, and (b) we subjected the CD45^–^ enriched cell fraction to a second MACS LS column. Cells were enumerated using a hemacytometer. Cell fractions throughout the procedure were analyzed for cell depletion and enrichment of CD45^–^ cells by flow cytometry by staining with fixable LIVE/DEAD dye (Invitrogen, L34955) and antibodies against the following cell surface antigens: CD45 (30-F11), CD31 (clone 390), PDPN (8.1.1), B220 (RA3-6B2), TCRβ (H57-597), CD11b (M1/70), and Ly6C (HK1.4) obtained from BioLegend. Following staining, cells were washed and fixed in 1% paraformaldehyde (PFA)/1% FBS, and data were acquired on a BD LSR Fortessa X-20 flow cytometer. Data analysis was performed using FlowJo analysis software (Tree Star Inc.).

### Single-cell library preparation.

Cells were subjected to single-cell droplet-encapsulation using the Next GEM Chip G Kit (catalog 1000127) and a 10X Genomics chromium controller housed in a BSL3 laboratory. We targeted recovery of 10,000 cells per replicate. Single-cell gene expression libraries were generated using the Next GEM single-cell 30 GEM library and gel bead kit v3.1 (catalog 1000128) and single index kit T set A (catalog 1000213). Sequences were generated with an Illumina NovaSEQ 6000 instrument using S4 flow cells and 300 cycle SBS reagents. We targeted 50,000 reads per cell, with the following sequencing parameters: Read 1, 151 cycles; i7 index, 8 cycles; i5 index, 0 cycles; Read 2, 151 cycles in accordance with the Chromium Next GEM single cell 3’ Reagent Kit v3.1 from 10X Genomics.

### CHIKV-specific library enrichment.

The scRNA-Seq libraries for the 8-hour time point were enriched for molecules aligning to the CHIKV genome according to a previously published method (34, 67). Specifically, the CHIKV genome was PCR amplified in 3 fragments (primer sequences: CHIKV-F1, 5′-TGAGACACACGTAGCCTACCA-3′; CHIKV-F2, 5′-AAGTCCAAGGGAATACAGATCTTC-3′; CHIKV-F3, 5′-ACCGCAGCACGGTAGAGA-3′; CHIKV-R1, 5′-CGAATAACATTACCTTGGAGCA-3′; CHIKV-R2, 5′-TTTTTCCCGGCCTATCACAG-3′; CHIKV-R3 5′-AAAAACAAAATAACATCTCCTACGTC-3′) and labeled with biotin-dUTP using the same primers before sonicating to generate ~150 bp fragments for hybridization. Denatured and diluted biotin-dUTP–labeled (MilliporeSigma) CHIKV genome fragments were hybridized to the concentrated scRNA-Seq libraries separately. Streptavidin capture beads (Invitrogen) were mixed with the hybridized libraries and washed to remove unbound DNA. Libraries were amplified directly from the cleaned-up beads and sequenced. FASTQ files for each replicate were processed using the cellranger count pipeline (v5.0.1). Reads were aligned to the mm10 and CHIKV AF15561 (EF452493.1) reference genomes.

To quantify CHIKV RNA levels for each cell identified in the enriched library, we calculated a CHIKV score (Figure 1, A and C), which is the number of CHIKV reads aligning to the CHIKV genome divided by the total mouse reads and CHIKV reads for each cell. To visualize this metric on UMAP (Figure 1A), a pseudo count (smallest nonzero value/2) was added to each value plotted.

### scRNA-Seq gene expression analysis.

FASTQ files for each replicate were processed using the Cell Ranger count pipeline (v5.0.1). Reads were aligned to the mm10 and CHIKV AF15561 (EF452493.1) reference genomes. Initial filtering of gene expression data was performed separately for the 8-hour and 24-hour time points using the Seurat R package (v4.2.0). Gene expression data for each biological replicate were combined into a single Seurat object. CHIKV reads were excluded from the gene expression matrices so they would not influence downstream processing (dimensionality reduction, clustering) of the mouse expression data.

Previously published scRNA-Seq data for the 24-hour time point was processed as previously described (27). CHIKV-low and -high cells were identified by filtering cells to only include those with > 5 CHIKV reads. K-means clustering was then used to independently group each biological replicate into CHIKV-low and -high populations. Cells with 5 CHIKV reads or fewer were included in the CHIKV-low population. Cells were filtered based on the number of detected mouse genes (>250 and <6,000) and the percent mitochondrial reads (<20%). Genes were filtered to only include those detected in > 5 cells. Potential cell doublets were removed using the DoubletFinder (v2.0.3) R package using an estimated doublet rate of 10%. Due to the ability of CHIKV to inhibit host transcription, CHIKV RNA–high cells with a low number of detected mouse genes (<250) or a high fraction of mitochondrial reads (>20%) were not filtered and remained in the data set for downstream analysis. The sgRNA ratio (Supplemental Figure 2, B, D, and E) was calculated by dividing the number of sgRNA (position 7567–12036) reads by the number of 5′ (position 1–7566) reads. To visualize this metric on UMAP (Supplemental Figure 2B), before calculating the sgRNA ratio, a pseudo count of 1 was added to the sgRNA and 5′ counts for each cell plotted (to eliminate division by 0).

Cells from the 8-hour time point samples were filtered based on the number of detected mouse genes (>250 and <8,000) and percentage mitochondrial reads (<20%). Genes were filtered to only include those detected in > 5 cells. The cell calls made by the cellranger pipeline (10X Genomics) for the second biological replicate for the 8-hour CHIKV time point were not accurate based on analysis of unique molecular identifier (UMI) counts and likely included a substantial number of empty droplets. To account for this, a cutoff of 800 UMI counts was used to remove potential empty droplets. Counts from the CHIKV-capture libraries were then added to the object for all cells passing our filtering cutoffs. Due to the very few cells with detectable CHIKV RNA at 8 hours, CHIKV^+^ cells were classified as any cell with at least 1 CHIKV-capture read aligning to the CHIKV genome.

For both the 8-hour and 24-hour time points, mouse gene expression reads were normalized by the total mouse reads for the cell, multiplied by a scale factor of 10,000, and log-transformed (NormalizeData). Normalized mouse counts were scaled and centered (ScaleData) using the top 2,000 variable features (FindVariableFeatures). The scaled data were used for PCA (RunPCA), and the first 40 principal components were used to identify clusters (FindNeighbors, FindClusters) and calculate UMAP (RunUMAP).

To ensure accurate and consistent cell type annotations, we integrated the 8-hour and 24-hour data sets based on time point and sample (mock- and WT CHIKV–infected) using the R package Harmony (v0.1.1) (68). We then reclustered the cells using the integrated data and generated an initial set of broad cell type annotations using the R package clustifyr (v1.8.0) (69) and reference data from Immgen (70). These annotations were checked for accuracy and further refined using known cell type markers, including *Cd19* (B cells), *Cd3e* (T cells), *Hbaa1* (erythrocytes), *Pdpn*, and *Pecam1*. To identify PvCs, fibroblasts were reclustered and integrated, and clusters were annotated using published reference data (20). To identify endothelial cell subsets, endothelial cells were reclustered and integrated, and clusters were annotated using published reference data (15). LEC and BEC annotations were further refined using known marker genes including *Marco*, *Pdpn*, and *Pecam1*. Visualization of integrated UMAP suggest that broad LN cell type and endothelial cell type annotations were consistent across conditions (Supplemental Figure 2, A and B). In addition, a strong correlation with the published reference data (Supplemental Figure 2C) and expression of key endothelial cell marker genes (Supplemental Figure 2D) further support the accuracy of our cell type annotations.

Differentially expressed genes were identified for each cell type for mock versus 8 hours and 8 hours versus 24 hours using the Seurat package. To allow for equal comparison with the 8-hour time point, the top 2 replicates (based on cell number) from the 24-hour time point were used for identifying differentially expressed genes. Genes were considered upregulated if the average log_2_ fold change was > 0.15 for 8 hours and > 0.25 for 24 hours for all replicates and the largest *P* value for all replicates was < 0.05. GO terms (Biological Process) were identified for the top 200 upregulated genes (sorted by maximum *P* value for replicates) for each cell type using the R package clusterProfiler (v4.4.4) (71). Terms were filtered to only include those with an adjusted *P* < 0.05 and at least 3 or 10 upregulated genes overlapping the term for the 8 hours and 24 hour time points, respectively. Terms with < 10 or > 750 genes were excluded from the analysis. Terms identified for each cell type were combined and clustered into 5 modules based on the pairwise overlap between terms using the clusterProfiler package. Enrichment scores were calculated by dividing the fraction of upregulated genes overlapping the term by the fraction of background genes overlapping the term. For Figure 5, C and E, cell types are only shown if they have at least 1 upregulated gene overlapping any of the terms plotted.

### Confocal microscopy.

LNs were fixed in periodate-lysine-paraformaldehyde (PLP) buffer containing 0.1 M L-lysine (MilliporeSigma), 2% PFA, and 2.1 mg/mL NaIO_4_ (MilliporeSigma) at pH 7.4 for 24–48 hours at 4°C, followed by incubation for 24 hours in 30% sucrose phosphate-buffered solution. Tissues were embedded in optimal cutting temperature (OCT) medium (Electron Microscopy Sciences), oriented to allow for sectioning through both the B cell follicles and the medullary sinuses, and frozen in dry-ice–cooled isopentane (MilliporeSigma). In total, 16 μm sections were cut on a Leica cryostat (Leica Microsystems). Sections were blocked with 5% goat, donkey, bovine, rat, or rabbit serum and then stained with 1 or more of the following antibodies: ERTR-7 (rat monoclonal, ERTR7, Abcam), B220 (RA3-6B2, Thermo Fisher Scientific), Lyve1 (ALY7, Thermo Fisher Scientific), CD11b (M1/70, BioLegend), CD36 (HM36), and/or MARCO (MCA1849, Serotec). Sections were incubated with secondary antibodies alone as controls, and images were acquired using identical photomultiplier tube (PMT) and laser settings. Images were acquired on a Leica SP8 or Stellaris confocal microscope (Leica Microsystems) using a 40× 1.3 NA or 63× 1.4 NA objective and merged to cover the entire LN using the Leica tilescan function. Images were processed and analyzed using Imaris software 8.0 (Oxford Instruments).

### Isolation of cells from LNs and flow cytometry.

LNs were gently homogenized in a Biomasher II tissue homogenizer (Kimble Chase) in RPMI 1640 (HyClone) supplemented with 5% FBS. For LN stromal cell isolation, left and right popliteal LNs from 2 mice were combined for each sample (4 LNs total), minced in Click’s media (Sigma-Aldrich) with 22G needles (Exelint), and digested for 1 hour at 37°C in 94 μg/mL DNase I (Roche) and either 250 μg/mL Liberase DL (Sigma-Aldrich) or 250 μg/mL collagenase type I and 250 μg/mL collagenase type IV (Worthington Biochemicals). Cell suspensions were passed through a 100 μm cell strainer (BD Falcon), and total viable cell numbers were enumerated by trypan blue exclusion. All single-cell suspensions were incubated for 15 minutes at 25°C in LIVE/DEAD Fixable Violet Dead Cell Stain (Invitrogen) to identify viable cells and then stained for 45 minutes at 4°C with anti–mouse FcγRIII/II (2.4G2; BD Pharmingen) and the following antibodies from BioLegend diluted in FACS buffer (PBS with 2% FBS): anti-CD45 (30-F11), anti-CD11b (M1/70), anti-CD11c (N418), anti-Ly6C (HK1.4), anti-Ly6G (1A8), anti-CD31 (clone 390), anti-PDPN (8.1.1), anti-CD36 (HM36), anti-CD206/MRC1 (C068C2), anti-CD41/ITGA2B (MWReg30), and anti-CD54/ICAM1 (YN1/1.7.4). Cells were washed 3 times in PBS/2% FBS and then fixed for 15 minutes in 1× PBS/1% PFA and analyzed on a BD LSR Fortessa cytometer using FACSDiva software. Analysis was performed using FlowJo software (Tree Star Inc.).

### Gene expression analysis by qPCR.

LNs were homogenized in TRIzol reagent (Invitrogen). RNA was isolated using the PureLink RNA Mini kit (Thermo Fisher Scientific) with on-column DNase treatment. Gene expression was quantified by qPCR using TaqMan gene expression assays (Thermo Fisher Scientific). Expression of each gene was normalized to 18S and analyzed as fold change over mock samples.

### Antigen acquisition by LNSCs.

Antigen acquisition was evaluated using fluorescently labeled ova as previously described (24). Ova (A5503, Sigma-Aldrich) was decontaminated of LPS using a Triton X-114 detoxification method and tested with Pierce LAL chromogenic endotoxin quantitation kit (88282, Thermo Fisher Scientific). Ova was labeled using an Alexa Fluor 488 succimidyl ester labeling system (A20100, Thermo Fisher Scientific). Mice were inoculated with 20 μg Alexa Fluor 488–labeled ova via intramuscular injection into both calf muscles (10 μg per calf), and popliteal and iliac LNs were collected for analysis of ova^+^ LNSCs by flow cytometry.

### Statistics.

Nonsequencing data were analyzed using GraphPad Prism version 10.1.1. software. Data were evaluated for statistically significant differences using a 2-tailed, unpaired *t* test, and either a 1-way or 2-way ANOVA test followed by Tukey’s multiple-comparison test. *P* < 0.05 was considered statistically significant.

### Study approval.

Animal experiments were performed with the approval of the IACUC of the University of Colorado School of Medicine (assurance no. A3269-01) under protocol no. 00026.

### Data availability.

The scRNA-Seq data underlying Figures 1 and 5 and Supplemental Figures 2 and 3 are available at NCBI GEO (GSE174667 and GSE243638). An analysis pipeline is available at https://github.com/rnabioco/morrison-lnsc (commit ID 20d5b7a508efc45ab96db525fe9a613c10b59154). Differentially expressed genes and GO terms for Figure 5 are shown in Supplemental Table 1. Values for all data points in graphs are reported in the Supporting Data Values file.

## Author contributions

CJL and TEM conceptualized the study. CJL, RMS, GVR, BJD, MKM, and AM conducted experiments and acquired the data. CJL, RMS, GVR, MKM, HDH, and TEM analyzed the data. BAJT and TEM provided resources. CJL and TEM wrote the original draft of the manuscript. CJL, RMS, JRH, HDH, BAJT, and TEM reviewed and edited the manuscript. JRH, BAJT, HDH, and TEM acquired funding. JRH, BAJT, and TEM supervised the study.

## Supplementary Material

Supplemental data

Supplemental table 1

Supporting data values

## Version 1 01/09/2024

In-Press Preview

## Version 2 02/22/2024

Electronic publication
