# Supplementary material for: Chikungunya virus infection disrupts lymph node lymphatic endothelial cell composition and function via MARCO
Source: JCI Insight. 2024 Jan 9;9(4):e176537. doi: 10.1172/jci.insight.176537 (PMC11143926; doi:10.1172/jci.insight.176537)
Supplement: Supplemental data [file jciinsight-9-176537-s125.pdf]

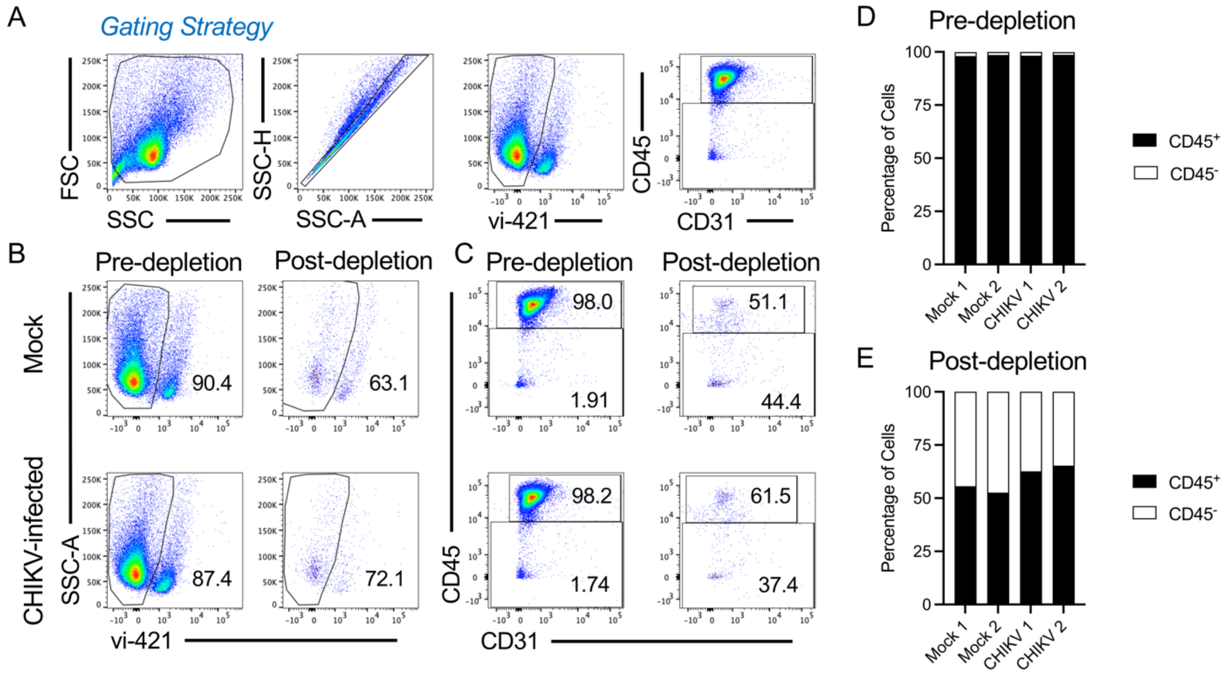


**Figure S1. Enrichment of CD45⁻ LN stromal cells for scRNA-seq.** (**A-E**) WT C57BL/6 mice were mock-inoculated (n = 2) or inoculated in both rear footpads with $10^3$ PFU CHIKV (n = 2). The left and right popliteal LNs were collected at 8 h post-infection for enrichment of $CD45^-$ LN stromal cells (LNSCs) via depletion of $CD45^+$ cells. The proportion of $CD45^+$ and $CD45^-$ cells was evaluated pre- and post-depletion by flow cytometry. (**A**) Representative flow cytometry plots showing the gating strategy for live $CD45^-$ LNSCs. (**B and C**) Representative flow cytometry plots of live cell viability (**B**) and percentage of $CD45^-$ cells (**C**) in mock and CHIKV-infected samples pre- and post-$CD45^+$ cell depletion. (**D and E**) Percentage of $CD45^+$ and $CD45^-$ cells in each condition and replicate pre- (**D**) and post-(**E**) $CD45^+$ cell depletion.

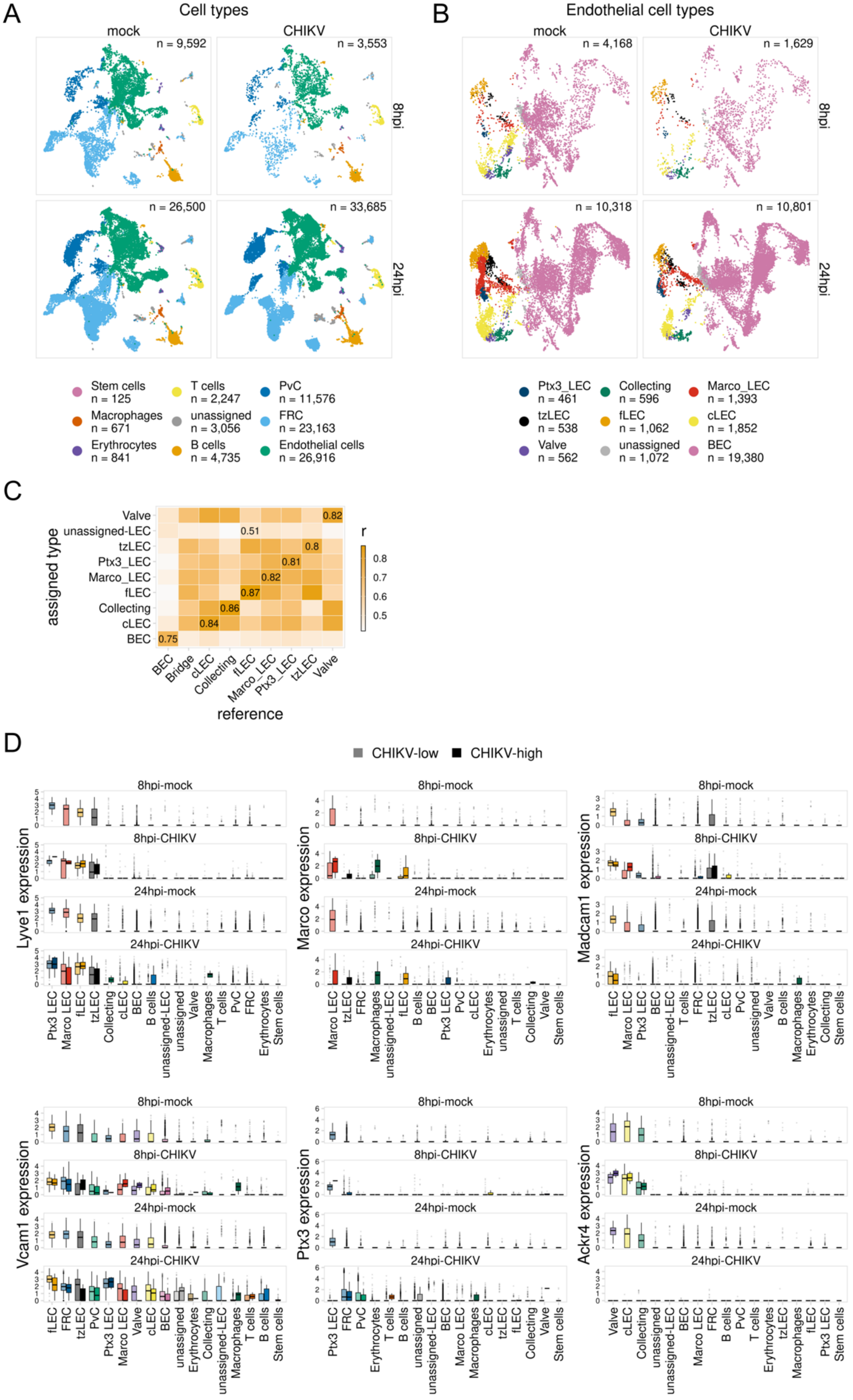
A
Cell types
mock
CHIKV
n = 9,592
n = 3,553
8hpi
n = 26,500
n = 33,685
24hpi
Stem cells n = 125
T cells n = 2,247
PvC n = 11,576
Macrophages n = 671
unassigned n = 3,056
FRC n = 23,163
Erythrocytes n = 841
B cells n = 4,735
Endothelial cells n = 26,916
B
Endothelial cell types
mock
CHIKV
n = 4,168
n = 1,629
8hpi
n = 10,318
n = 10,801
24hpi
Ptx3_LEC n = 461
Collecting n = 596
Marco_LEC n = 1,393
tzLEC n = 538
fLEC n = 1,062
cLEC n = 1,852
Valve n = 562
unassigned n = 1,072
BEC n = 19,380
C
assigned type
Valve
unassigned-LEC
tzLEC
Ptx3_LEC
Marco_LEC
fLEC
Collecting
cLEC
BEC
0.82
0.51
0.8
0.81
0.82
0.87
0.86
0.84
0.75
r
0.8
0.7
0.6
0.5
BEC
Bridge
cLEC
Collecting
fLEC
Marco_LEC
Ptx3_LEC
tzLEC
Valve
reference
D
CHIKV-low
CHIKV-high
8hpi-mock
8hpi-CHIKV
24hpi-mock
24hpi-CHIKV
Lyve1 expression
Marco expression
Madcam1 expression
Vcam1 expression
Ptx3 expression
Ackr4 expression

**Figure S2. Cell type annotation of scRNA-seq data.** WT C57BL/6 mice were inoculated with PBS (mock, n = 3) or $10^3$ PFU of CHIKV (n = 3) in the left-rear footpad. At 8 and 24 h post-infection, the dLN was collected and enzymatically digested into a single-cell suspension. Cells were enriched for $CD45^-$ cells and analyzed by scRNA-seq as previously described (34). (**A**) UMAP projections of cell type annotations for integrated data. (**B**) UMAP projections of endothelial cell type annotations for integrated data. (**C**) Correlation between annotated LEC subsets and reference data. (**D**) Expression of select marker genes across LN cells.

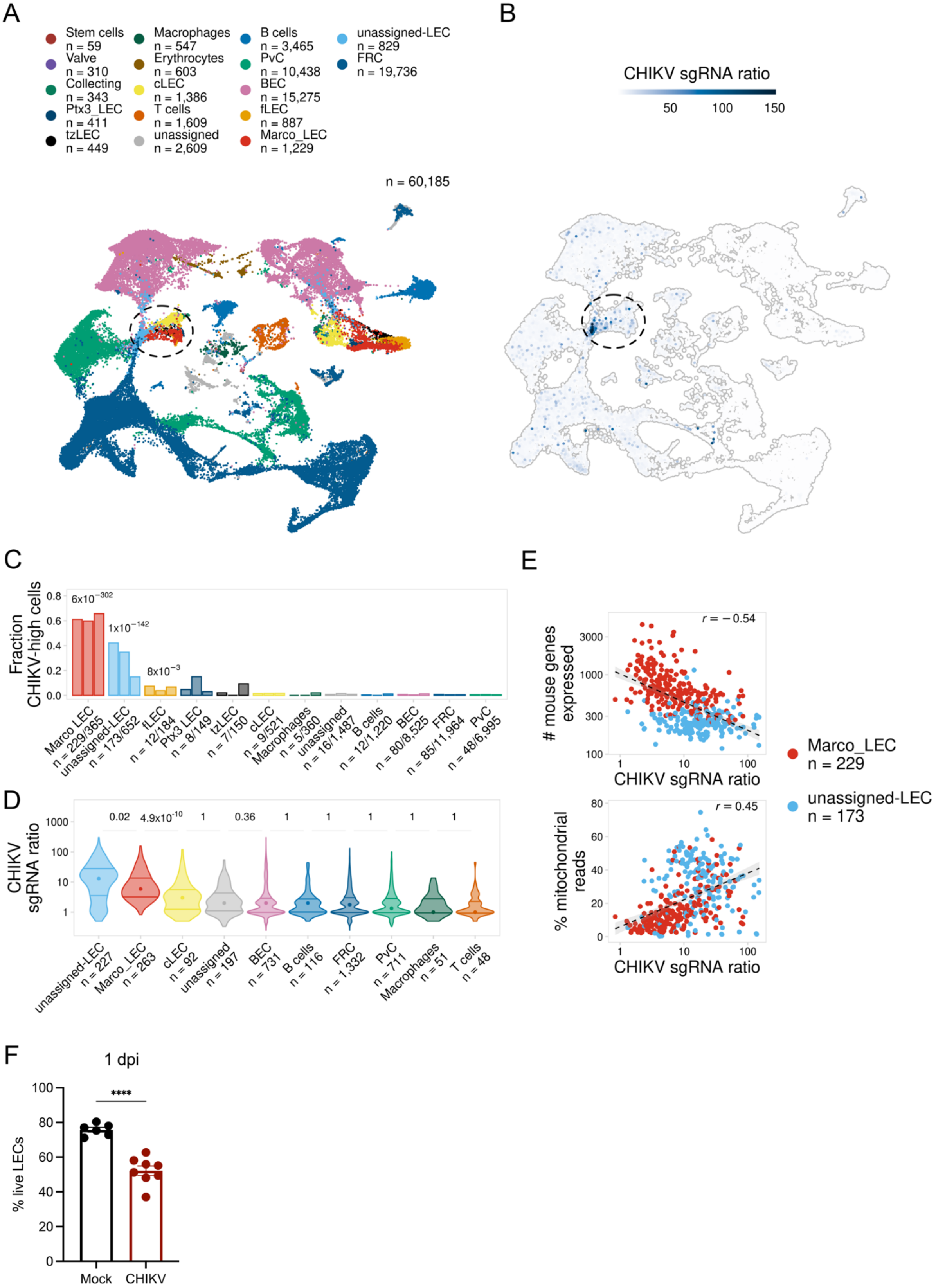
A
Stem cells n = 59
Valve n = 310
Collecting n = 343
Ptx3_LEC n = 411
tzLEC n = 449
Macrophages n = 547
Erythrocytes n = 603
cLEC n = 1,386
T cells n = 1,609
unassigned n = 2,609
B cells n = 3,465
PvC n = 10,438
BEC n = 15,275
fLEC n = 887
Marco_LEC n = 1,229
unassigned-LEC n = 829
FRC n = 19,736
n = 60,185
B
CHIKV sgRNA ratio
50 100 150
C
Fraction CHIKV-high cells
6x10−302
1x10−142
8x10−3
Marco LEC n = 229/365
unassigned-LEC n = 173/652
fLEC n = 12/184
Ptx3 LEC n = 8/149
tzLEC n = 7/150
cLEC n = 9/521
Macrophages n = 5/360
unassigned n = 16/1,487
B cells n = 12/1,220
BEC n = 80/8,525
FRC n = 85/11,964
PvC n = 48/6,995
D
CHIKV sgRNA ratio
0.02 4.9x10-10 1 0.36 1 1 1 1 1
unassigned-LEC n = 227
Marco_LEC n = 263
cLEC n = 92
unassigned n = 197
BEC n = 731
B cells n = 116
FRC n = 1,332
PvC n = 711
Macrophages n = 51
T cells n = 48
E
# mouse genes expressed
r = −0.54
CHIKV sgRNA ratio
% mitochondrial reads
r = 0.45
CHIKV sgRNA ratio
Marco_LEC n = 229
unassigned-LEC n = 173
F
1 dpi
% live LECs
****
Mock CHIKV

**Figure S3. Signs of CHIKV RNA replication in MARCO-expressing LECs.** (**A-E**) WT C57BL/6 mice were inoculated with PBS (mock, n = 3-6) or $10^3$ PFU of CHIKV (n = 3-8) in the left-rear footpad. At 24 h post-infection, the dLN was collected and enzymatically digested into a single-cell suspension. Cell suspensions were either enriched for $CD45^-$ cells and analyzed by scRNA-seq as previously described (34) or analyzed for cell viability by flow cytomtery. (**A**) UMAP projection shows annotated cell types. (**B**) UMAP projection shows CHIKV sgRNA ratio (number of sgRNA reads/number of 5' reads). (**C**) The fraction of cells identified as CHIKV-high is shown for each cell type. Labels show the number of CHIKV-high cells/total cells. P values were calculated as described in **Figure 1D**. (**D**) CHIKV sgRNA ratio for cells with >0 sgRNA reads and >0 5' reads. Only cell types with >40 cells are shown. P values were calculated using a two-sided Wilcoxon rank sum test with Bonferroni correction. (**E**) The correlation between CHIKV sgRNA ratio and QC metrics for CHIKV-high $MARCO^+$ LECs and unassigned-LECs. (**F**) LN LEC viability at 1 d post-infection was determined by flow cytometric analysis of cell populations stained with a live-dead cell viability dye. ****, $P < 0.000$, student's t-test (2 independent experiments).

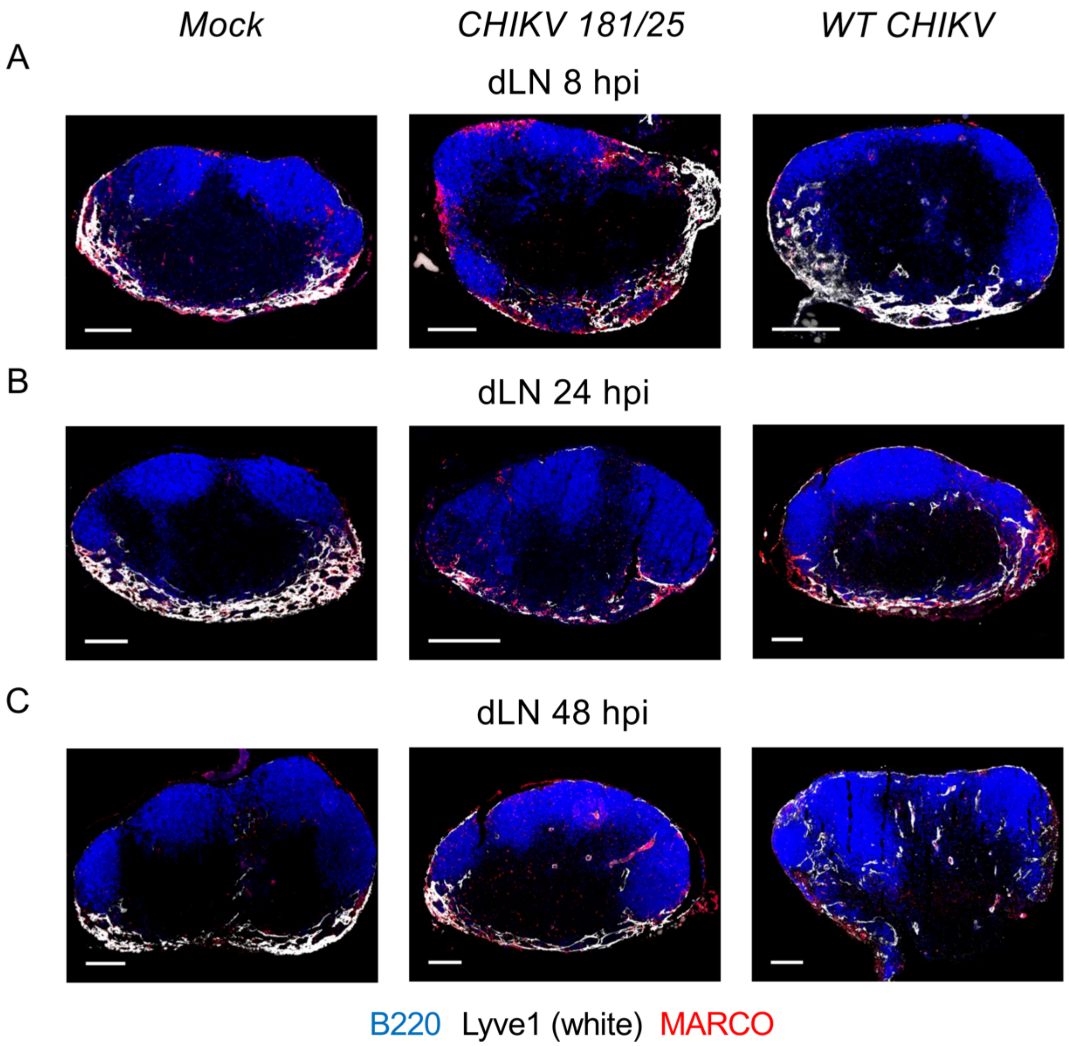


**Figure S4. Lyve-1 and MARCO expression over time during WT and attenuated CHIKV infection.** (**A-C**) WT C57BL/6 mice were mock-inoculated (n = 3) or inoculated in the footpad with $10^3$ PFU CHIKV 181/25 (n = 5) or WT CHIKV (n = 5). At 8 (**A**), 24 (**B**), or 48 (**C**) h post-infection the dLN was collected. Frozen dLN sections were stained for B220 (B cells; blue), Lyve-1 (LECs; white), and MARCO (red). Scale bar, 200 μm. Images are representative of 3-5 dLNs per group (2 independent experiments).

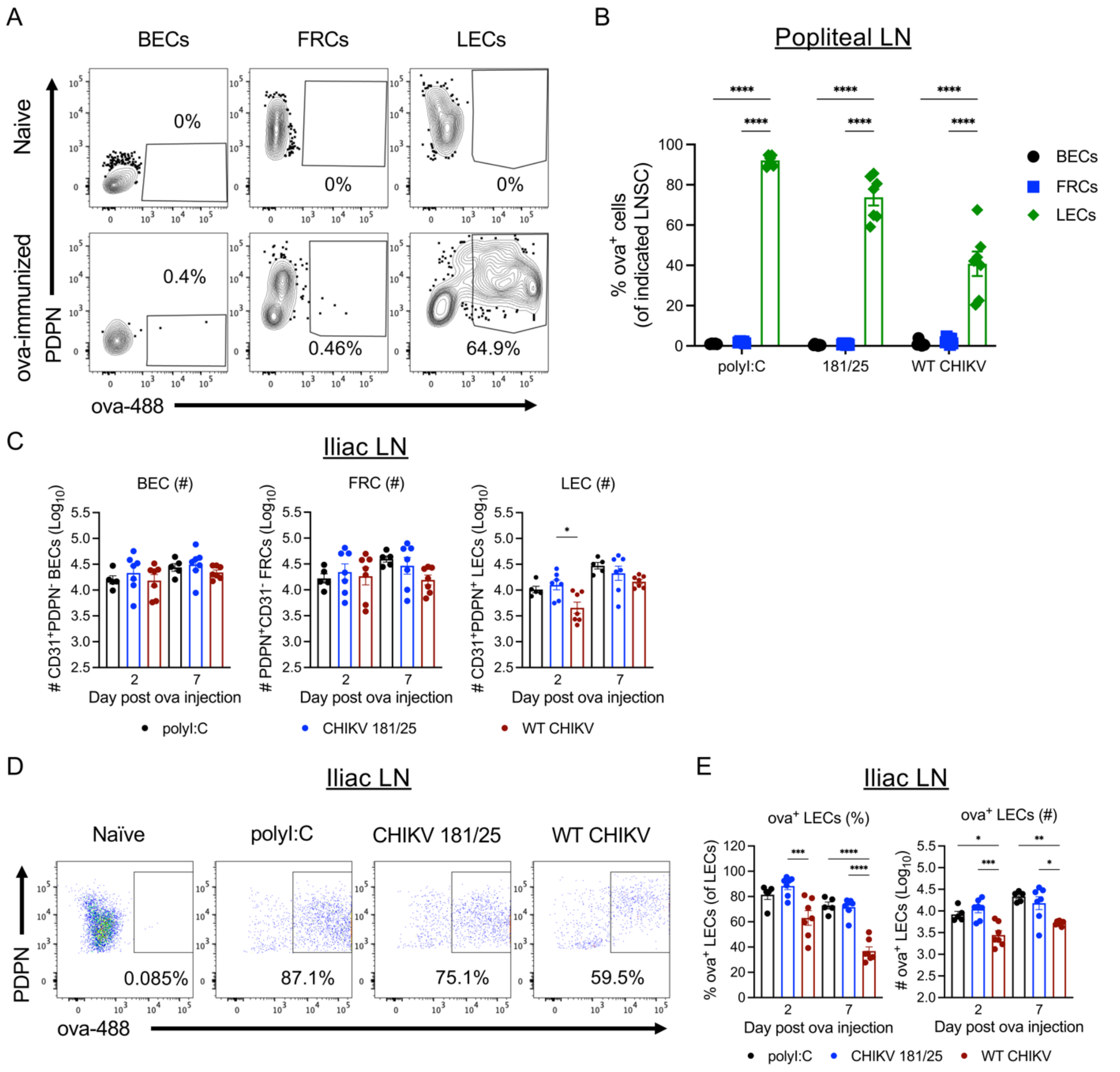


**Figure S5. Impaired antigen acquisition is LEC-specific and not limited to the popliteal LN.** WT C57BL/6 mice were mock-infected or infected in the footpad with $10^3$ PFU CHIKV 181/25 or WT CHIKV. At 72 h post-infection, mice were inoculated with 10 µg ova-488 in both calf muscles (20 µg total), and ova$^+$ LNSCs in the popliteal and iliac LNs were then evaluated by flow cytometry at the indicated timepoints. As a positive control, naïve mice were injected with 10 µg ova-488 and 5 µg polyI:C in both calf muscles. Representative plots showing ova$^+$ LNSCs in the popliteal LN (**A**). Percentage of ova$^+$ BECs, FRCs, and LECs among each condition in the popliteal LN.

(**B**) LNSC numbers in the iliac LN following ova immunization (**C**). Representative plots showing ova$^+$ LECs in the iliac LN, including the naïve control for gating on ova$^+$ LECs (**D**) and quantification of percentage and number of ova$^+$ LECs (**E**). Only statistical comparison of ova$^+$ BECs, FRCs, and LECs within each condition is shown. ***, $P < 0.01$; ****, $P < 0.0001$, one or two-way ANOVA with Tukey's multiple comparison test (2 independent experiments).
